# Differential abundance of microRNAs in seminal plasma extracellular vesicles (EVs) in Sahiwal cattle bull related to male fertility

**DOI:** 10.3389/fcell.2024.1473825

**Published:** 2024-10-01

**Authors:** Vitika Chauhan, Poonam Kashyap, Jatinder Singh Chera, Ankit Pal, Aditya Patel, Seema Karanwal, Shiva Badrhan, Fanny Josan, Subhash Solanki, Mukesh Bhakat, Tirtha Kumar Datta, Rakesh Kumar

**Affiliations:** ^1^ Animal Genomics Laboratory, Animal Biotechnology Division, National Dairy Research Institute, Karnal, India; ^2^ Livestock Production and Management Division, ICAR- Central Institute of Research on Goat, Mathura, Uttar Pradesh, India; ^3^ Central Institute for Research on Buffaloes, Hisar, Haryana, India

**Keywords:** Sahiwal, seminal plasma extracellular vesicles, RNA-seq, miRNA, bull fertility

## Abstract

Sahiwal cattle, known for their high milk yield, are propagated through artificial insemination (AI) using male germplasm, largely contingent on semen quality. Spermatozoa, produced in the testes, carry genetic information and molecular signals essential for successful fertilization. Seminal plasma, in addition to sperm, contains nano-sized lipid-bound extracellular vesicles (SP-EVs) that carry key biomolecules, including fertility-related miRNAs, which are essential for bull fertility. The current study focused on miRNA profiling of SP-EVs from high-fertile (HF) and low-fertile (LF) Sahiwal bulls. SP-EVs were isolated using size exclusion chromatography (SEC) and characterized by dynamic light scattering (DLS) and nanoparticle tracking analysis (NTA). Western blotting detected the EV-specific protein markers TSG101 and CD63. The DLS analysis showed SP-EV sizes of 170–180 nm in HF and 130–140 nm in LF samples. The NTA revealed particle concentrations of 5.76 × 10^10^ to 5.86 × 10^11^ particles/mL in HF and 5.31 × 10^10^ to 2.70 × 10^11^ particles/mL in LF groups, with no significant differences in size and concentration between HF and LF. High-throughput miRNA sequencing identified 310 miRNAs in SP-EVs from both groups, with 61 upregulated and 119 downregulated in HF bull. Further analysis identified 41 miRNAs with significant fold changes and p-values, including bta-miR-1246, bta-miR-195, bta-miR-339b, and bta-miR-199b, which were analyzed for target gene prediction. Gene Ontology (GO) and KEGG pathway analyses indicated that these miRNAs target genes involved in transcription regulation, ubiquitin-dependent endoplasmic reticulum-associated degradation (ERAD) pathways, and signalling pathways. Functional exploration revealed that these genes play roles in spermatogenesis, motility, acrosome reactions, and inflammatory responses. qPCR analysis showed that bta-miR-195 had 80% higher expression in HF spermatozoa compared to LF, suggesting its association with fertility status (*p* < 0.05). In conclusion, this study elucidates the miRNA cargoes in SP-EVs as indicators of Sahiwal bull fertility, highlighting bta-miR-195 as a potential fertility factor among the various miRNAs identified.

## Introduction

High genetic merit bulls have the capacity to propagate their progenies at a faster rate, as a single bull can be used to breed thousands of cows through artificial insemination (AI). The availability of good breeding bulls is a major bottleneck in the production of the requisite quantity of frozen straws. Limitation of producing sufficient number of frozen straws remains a concern in Sahiwal cattle farming. While both males and females contribute to the success or failure of pregnancy, the selection of highly fertile bulls holds greater significance in artificial breeding. Although bulls are selected based on breeding soundness evaluation (BSE) criteria, bull fertility remains diverse in bull farms (Tanga et al., 2021). A single ejaculate from a bull contains only 5% sperm and 95% seminal plasma. Seminal plasma is a blend of fluids produced by different glands and sexual organs, serving as a protective environment for sperm following ejaculation (Juyena and Stelletta, 2012; Druart and de Graaf, 2018). Seminal plasma plays a crucial role in sperm metabolism, function, survival, and transport. This indicates significant interactions and material exchanges occur between seminal plasma and sperm (Björndahl and Kvist, 2009; Bromfield, 2014). Additionally, seminal plasma is rich in various forms of RNA, including mRNA, microRNA, and tsRNAs (Li et al., 2012; Kuster and Althouse, 2016). The seminal plasma of bulls contains a large number of extracellular vesicles (SP-EVs), similar to that found in other animals (Talluri et al., 2017). The seminal plasma EVs are small, nano-sized (50–500 nm), lipid membrane-bound structures that are released mainly by the epididymis and the prostate (Saez et al., 2003; Aalberts et al., 2014). EVs transport different types of cargo like DNA, coding and non-coding RNA (miRNA), proteins, lipids, and carbohydrates (Takahashi et al., 2017) to the spermatozoa. EVs help in cell-cell communication and regulate sperm functions (Machtinger et al., 2016).

SP-EVs play a pivotal role in different sperm functions, such as sperm capacitation (Pons-Rejraji et al., 2011), sperm motility (Fabiani et al., 1995; Murdica et al., 2019), and the acrosome reaction (Piehl et al., 2013). Non-coding RNAs (nc-RNAs) in seminal plasma extracellular vesicles have been linked to the protection of sperm within the female genital tract (Aalberts et al., 2014) and also help in sperm-egg fusion (Franchi et al., 2020). The repertoire of non-coding RNAs (nc-RNAs), particularly microRNAs (miRNAs), and proteins in human semen extracellular vesicles (EVs) was analyzed using RNA and proteome sequencing. miRNAs are single-stranded non-coding RNA molecules, typically 18–25 base pairs in length, that regulate gene expression at the post-transcriptional level (Iacomino, 2023). miRNAs play a role in regulating numerous genes associated with reproductive processes, including the development and function of the reproductive tract, germ cell development and maturation, fertilization, and early embryonic development (Vojtech et al., 2014). The abundance of miRNAs in semen-derived extracellular vesicles (EVs) significantly increased from 7.5% in the caput epididymis to 31% in the cauda epididymis, suggesting that miRNAs in seminal plasma EVs played a crucial biological role (Chen et al., 2020). Using high-throughput sequencing, a study of the expression of miRNAs in the testes of chickens with low and high sperm motility revealed that 182 miRNAs were upregulated and 120 miRNAs were downregulated in the testes of chickens with high motility sperm (Xing et al., 2020). It also showed that has-miR-629-3p is linked to sperm motility in human seminal samples (Salas-Huetos et al., 2015), and that miRNA-122-5p plays a key role in sperm motility and the ability of sperm to fertilize an oocyte in bull sperm (da Silva et al., 2021). Furthermore, the expression of let-7a, let-7d, let-7e, and miR-22 was increased in porcine sperm with abnormal motility and morphology, while the expression of miR-15b was decreased (Curry et al., 2011). However, the mechanism by which miRNAs affect sperm motility and morphology has not yet been explored. SP-EVs are rich in bioactive molecules, including proteins and small non-coding RNAs (sncRNAs) such as miRNAs, Piwi-interacting RNAs (piRNAs), ribosomal RNAs (rRNAs), and transfer RNAs (tRNAs) (Lange-Consiglio et al., 2022). miR-31-5p has been identified as a biomarker for azoospermia (Lange-Consiglio et al., 2022), while two exosomal miRNAs, miR-196-5p and miR-501-3p, are known to be downregulated in prostate cancer.

The miRNA from SP-EVs of the various animals, including rats (Fornés et al., 1991), rabbits (Davis, 1978), bulls (Breitbart and Rubinstein, 1982) stallions (Arienti et al., 1998), and boars (Piehl et al., 2013) have been reported. However, the full miRNA profile of SP-EVs in Sahiwal cattle bulls has not yet been investigated, and it remains unclear how the dynamics of miRNA in SP-EVs compared to spermatozoa affect the fertilizing potential of the sperm. Given this background, in the current study we investigated the differentially expressed short non-coding miRNA profiles of SP-EVs in Sahiwal bulls of distinct fertility status. We report the detection of these specific miRNAs in both SP-EVs and spermatozoa, providing a comprehensive understanding of how the transfer of miRNAs from SP-EVs to spermatozoa affects fertility in Sahiwal bulls. The findings from this study could offer valuable insights into the role of miRNAs cargo in SP-EVs in bull fertility.

## Materials and methods

### Selection of distinct fertility bulls and collection of fresh semen

The standing bulls included in this study were sourced from the Artificial Breeding Research Centre (ABRC) at the National Dairy Research Institute (NDRI), India, based on their fertility status. Out of the 32 bulls assessed, six Sahiwal bulls with distinct fertility, each having over 50 records of artificial inseminations, were selected for further investigation. These bulls underwent evaluation for breeding soundness and semen quality parameters, including semen volume, sperm count, viability, and progressive motility. The chosen bulls were managed in accordance with the routine feeding and management plan of ABRC, NDRI.

The conception rates (CR) of the 32 bulls were examined for normal distribution. The CR demonstrated a normal distribution with a mean (M) of 44.485% and a standard deviation (S.D.) of 7.88%. Bulls with CRs below M-1 S.D. (36.6%) were classified as low fertile (LF), while those with CRs above M+1 S.D. (52.36%) were classified as high fertile (HF) (Supplementary Table S1).

For this study, six Sahiwal bulls (n = 6) were selected, with three falling into each fertility category (HF, n = 3, CR between 55.81% and 59.59%, and LF, n = 3, CR between 29.15% and 30.43%) shown in Supplementary Figure S1 (Karanwal et al., 2023; Batra et al., 2020; Prakash et al., 2021; Somashekar et al., 2017).

### Isolation and processing of seminal plasma

The semen samples from HF (n = 3) and LF (n = 3) bulls were collected and transported to the lab at 37°C, ensuring no delay (within 30 min). They were then centrifuged at 1,520 g for 15 min. The resulting supernatant was transferred to a fresh tube and centrifuged again at 850 g for 5 min at 37°C. The final supernatant (seminal plasma) was either utilized for EV isolation or stored at −80°C (Badrhan et al., 2024).

### Isolation of SP-EVs through size exclusion chromatography

SEC columns were assembled using Sepharose CL-2B (100 mL, Sigma Aldrich; New Delhi, India) and rinsed with 1X PBS containing 0.32% trisodium citrate (pH 7.4, 0.22 μm filtered). The column was packed with 10 mL of Sepharose 2B beads. Subsequently, 1 mL of seminal plasma was applied to the column, and elution was carried out using PBS + 0.32% trisodium citrate. The eluted material was collected in 26 distinct fractions of 0.5 mL each (Badrhan et al., 2024; Böing et al., 2014). Each fraction was then subjected to dynamic light scattering and nanoparticle tracking analysis to determine size and concentration, following the methods described by Böing et al. (2014). The composite of various SEC fractions of extracellular vesicles (EVs) was employed for subsequent experiments (Böing et al., 2014; Pal et al., 2023).

### Characterization of SP-EVs

EVs were isolated from seminal plasma sample of three HF and LF bulls as previously described and pooled together. The isolated EVs were characterized through several approaches following MISEV 2018 guidelines.

### Differential light scattering

Using a Zetasizer Nano ZS ZEN3600 (Malvern Instruments, Malvern, United Kingdom), the size distribution of EVs was determined. The scattered light’s intensity was measured at 173°. The particle size measurement was made in triplicate at 25°C, followed by data analysis and processing, which were carried out with the help of the Zetasizer software, version 7.03. After EV isolation, 10 μL of purified EVs were diluted in 990 μL of filtered 1X PBS and sonicated in an ultrasonic water bath for 1 min at 50 Hz. The sample was immediately put in a disposable cuvette for size measurements to avoid aggregation of the EVs. Three independent measurements were recorded for each sample (Pal et al., 2023; Szatanek et al., 2017).

### Nanoparticle tracking assay

All particle tracking analyses used a Malvern NS300 device with a 488 nm laser and a 500 nm long-pass filter for fluorescence detection. To perform the NTA count, all samples were diluted in a ratio of 1:100 with 1X PBS (10010023, Gibco). The sample infusion pump was set to a constant flow rate of 5 μL/min. The camera level was set at 14, as all particles were visible at this level without signal saturation, and the detection threshold was set at 5. The ideal measurement concentration was found by pre-testing the ideal particles per frame value (40–120 particles/frame). The sample was measured five times, and 30- to 60-sec videos were collected with a minimum of 200 valid tracks recorded per video. Ten of the particles tracked were calculated and graphed using GraphPad Prism. All studies were performed on Nanosight 3.0 software using the default settings (Doyle and Wang, 2019; Pal et al., 2023).

### Transmission electron microscopy

Isolated EVs were diluted 1:100 in 1X PBS and fixed with an equal volume of 2% paraformaldehyde prepared in phosphate buffer for 1 h at 4°C. The carbon Formvar film-coated 300-mesh transmission electron microscopy grids were glow-discharged. The fixed EV samples were applied to the TEM grid and incubated for 15 min at room temperature. After washing with PBS, the samples were fixed with 1% glutaraldehyde for 5 min. Upon washing with distilled water, the grids were stained with 1% phosphotungstic acid for 45 s, wicked off with Whatman filter paper, and allowed to dry before viewing. TEM examination was performed using the JEM1400 FLASH transmission electron microscope (JEOL USA Inc., Peabody, MA, United States) at 120 kV and viewed under ×250,000 magnification with a highly sensitive Olympus sCMOS camera at the TEM facility of the Advanced Technology Platform Centre (ATPC), Regional Centre for Biotechnology, Faridabad (Pal et al., 2023).

### Western blotting

Protein was isolated by incubating the isolated EVs in RIPA buffer (Sigma-Aldrich) for 15 min at room temperature, followed by sonication. Approximately 20 µg of protein was loaded per well into SDS-PAGE gel, and proteins were separated by SDS polyacrylamide gel electrophoresis using a mini gel tank electrophoresis system (Invitrogen Life Technologies) and then transferred to Immobilon-FL polyvinylidene difluoride membranes (Millipore, Billerica, MA, United States). The membrane containing the transferred protein was probed with primary mouse polyclonal anti-TSG-101 (1:1000 SC-7964, Santa Cruz Biotechnology, United States), primary goat anti-CD63 (1:3000, STJ140029, St. Johns Laboratory, London, United Kingdom), primary CD9 monoclonal antibody (IVA50) (1:6000, MA1-19301, Invitrogen), and primary anti-Calnexin (CNX) monoclonal antibody (CAA280Hu22, Cloud Clone Corp, United States). Membranes were washed in Tris-buffered saline (TBS, pH 7.6) and incubated for 2 h in TBST (TBS with Tween-20) containing 5% BSA along with horseradish peroxidase-conjugated anti-mouse secondary antibody (1:10000, SC-516102, Santa Cruz Biotechnology), anti-goat secondary antibody (1:20000, STJ99512, St. Johns Laboratory), and anti-mouse secondary antibody (for CD9-1:30000, for Calnexin- 1:10000, A9044, Sigma), respectively. The Substrate Pierce ECL Western Blotting kit (32106) was utilized in the next step for chemiluminescence, and subsequently, the membranes were exposed to X-ray film for 1–5 min before visualization (Karanwal et al., 2023).

### RNA extraction from SP-EVs

Isolated SP-EV fractions (7–16) were pooled and suspended in TRIzol (Ambion, Thermo Fisher Scientific, United States). Then the phase separation was performed by adding 200 µL of chloroform, followed by an invert mix and centrifugation at 13,200 rpm for 15 min at 4°C. The aqueous phase was transferred to fresh microcentrifuge tubes, and RNA was precipitated overnight at −80°C with an equal volume of isopropanol and 1 µL (20 μg/μL) glycogen. The pellet recovered by centrifugation was washed with 70% ethanol. The air-dried pellet was re-suspended in nuclease-free water. The concentration and purity of RNA were quantified using the Nanodrop Spectrophotometer (Thermo Scientific, 2000).

### Library preparation

5 μL of the total RNA was taken for fragmentation and priming. Fragmented and primed RNA was further subjected to first-strand synthesis, followed by second-strand synthesis. The double-stranded cDNA was purified using NEBNext purification beads (NEBNext, Cat #E7767S). Purified cDNA was end-repaired, adenylated, and ligated to Illumina multiplex barcode adapters as per the NEBNext^®^ UltraTM II Directional RNA Library Prep protocol, followed by second-strand excision using the USER enzyme at 37°C for 15 min.

The Illumina Universal Adapters used in the study were:

5′AAT​GAT​ACG​GCG​ACC​ACC​GAG​ATC​TAC​ACT​CTT​TCC​CTA​CAC​GAC​GCT​CTT​CCG​ATC​T-3′ and Index Adapter 5′-GAT​CGG​AAG​AGC​ACA​CGT​CTG​AAC​TCC​AGT​CAC [INDEX] ATC​TCG​TAT​GCC​GTC​TTC​TGC​TTG-3' [INDEX] Unique sequence to identify sample-specific sequencing data. Adapter-ligated cDNA was purified using NEBNext purification beads and subjected to 12 cycles of indexing [98°C for 30 s, cycling (98°C for 10 s, 65°C for 75 s, and 65°C for 5 min)] to enrich the adapter-ligated fragments. The final PCR product (sequencing library) was purified with JetSeq beads, followed by a library quality control check (Prakash et al., 2021).

### RNA sequencing and data analysis

The libraries were paired-end sequenced on an Illumina Novaseq 6000 sequencer for 150 cycles (Illumina, San Diego, United States) following the manufacturer’s instructions. Transcriptome analysis was done by processing the raw data for the removal of low-quality reads and adapter sequences. Expression analysis was performed using the high-quality reads after alignment with the reference genome [*Bos indicus*] using a splice-aware aligner. The raw reads were processed using FastQC1 for quality assessment and pre-processing, which includes removing adapter sequences and low-quality bases (< q30) using TrimGalore2. The pre-processed, high-quality data was aligned to the *Bos indicus* reference genome downloaded from the NCBI database using Hisat2 with default parameters. Reads were classified as aligned (which align to the reference genome) and unaligned reads. The feature count tool was used to estimate and calculate transcript abundance. Absolute counts for transcripts were estimated and used in differential expression analysis.

DESeq25 was used to calculate the differentially expressed transcripts. It performs an internal normalization where the geometric mean is calculated for each transcript across all samples. It fits negative binomial generalized linear models for each transcript and uses the Wald test for significance testing. Further, the expressed transcripts were categorized into up, down, and neutrally regulated based on the log2fold change cutoff of ±1 value.

### miRNA target prediction

We employed pre-computed predictions from TargetScan (TS), miRmap (MM), microTCDS (mt), miRdb, and miRwalk, all of which are publicly accessible online. Target Scan, miRmap, takes into account the seed-based interactions in the target miRNA’s 3′UTR. Based on the miRTarget (MT) score determined by examining hundreds of miRNA target interactions from high-throughput sequencing research, targets in the miRDB are predicted. MicroTCDS recognizes miRNA targets in both the coding and 3′UTR regions (Van Rooij, 2011). These tools were chosen because their miRNA prediction capabilities were the most complete and they have an update strategy. In order to increase the sensitivity of target prediction for bta-miR-1246, bta-miR-195, and bta-miR-199b, we take into account the union of the minimum tools in the venn diagram of four tools for prediction. With the exception of bta-miR-339b, only three programs were used because this miRNA was not available in the database (Pal et al., 2023).

### Gene ontology and pathway enrichment analysis

Gene ontology analyses were conducted on the chosen miRNA target genes and classified as biological processes (BP), molecular functions (MF), and cellular components (CC) through the Database for Annotation, Visualization, and Integrated Discovery (DAVID) and Kyoto Encyclopedia of Genes and Genomes (KEGG) pathway enrichment analysis. Significance was determined for pathways and GO terms with the highest number of genes, employing a p-value threshold of 0.05 (Nguyen et al., 2019; Fan et al., 2022).

### Isolation of miRNA from sperm and cDNA synthesis

The miRNA was isolated from the sperm of HF (n = 3) and LF (n = 3) using the Qiagen Advanced miRNeasy Serum/plasma kit (cat. 217204, Qiagen, Germany) according to the manufacturer’s instructions, with the addition of bacteriophage MS2 RNA (10165948001, Roche) as a carrier RNA (to improve the RNA yield) in a 20 μL volume. Approximately 120 ng of the isolated RNA was converted into cDNA for each sample using the Reverse Transcriptase Kit II miScript (Cat# 218161 Qiagen, Valencia, CA) using HiSpec buffer (5X), miScript nucleic acid mix, and miScript enzyme (RT) in a final reaction volume of 20 µL for mature miRNA profiling. miRNA, cel-miR-39-3p, was used as an exogenous spike-in control. The RNA concentration for each sample was measured using a NanoDrop 1000 spectrophotometer (NanoDrop Technologies), and a 10–15 ng/μL concentration range was obtained from each sample. Approximately 120 ng of the isolated RNA was converted into cDNA for each sample using the Reverse Transcriptase Kit II miScript (Cat # 218161 Qiagen, Valencia, CA) using HiSpec buffer (5X), miScript nucleic acid mix, and miScript enzyme (RT) in a final reaction volume of 20 µL for mature miRNA profiling. The samples were incubated at 37°C for 1 h and then at 95°C for 5 min to inactivate the miScript enzyme (RT). Subsequently, the cDNA was diluted in nuclease-free water (2.5 ng/μL for qPCR) and stored at −20°C (Dubey et al., 2023; Pal et al., 2023).

### Primer design for miRNA

Specific miRNAs were selected based on the NGS data that indicated their highest abundance in high-fertile cattle. A total of four miRNAs, i.e., bta-miR-195, bta-miR-1246, miR-195-5p, and miR-200a-3p, were selected, and miR-26a, let-7a, and miR-16a were initially chosen as reference controls. Primers were designed using the miR primer tool (Busk, 2014). The forward primers were made against the seed sequence of the miRNAs from the database available at http://www.mirbase.org/. The reference controls miR-23a, miR-26a, and U6 were later excluded due to their lesser stability in different biological samples as calculated by the online tool RefFinder (http://www.heartcure.com.au/refnder/). The mature miRNAs and the primer sequences utilized for the q-PCR assay have been listed in Supplementary Table S14. Primer oligos were procured from Sigma-Aldrich (Pal et al., 2023).

### miRNA quantification using quantitative real-time PCR (RT-qPCR)

Relative quantification of selected miRNAs was performed using the miScript SYBR Green PCR Kit (QIAGEN, Valencia, CA, United States). Approximately 5 ng of cDNA was used as a template in each reaction. A no-template control (NTC) was also run on each plate. The reactions were performed in duplicate using the Bio-Rad c1000 thermal cycler detection system (United States) with the following conditions: 95°C for 5 min, 45 amplification cycles consisting of incubations at 94°C for 15 s, 55°C for 45 s, and 70°C for 30 s, followed by melt curve analysis from 65°C to 95°C. The relative expression of the target miRNAs was calculated using the 2−ΔΔCT method by considering let-7a and miR-16a as reference controls (Livak and Schmittgen, 2001). The differential expression levels of miRNAs between high-fertility and low-fertility bulls were analyzed statistically. The qPCR data was analyzed using GraphPad Prism 8.0. The datasets were analyzed by an unpaired t-test, and the difference was considered to be significant at P < 0.05 (Pal et al., 2023).

## Results

### Isolation and characterization of EVs from the seminal plasma of Sahiwal cattle bulls

**FIGURE 1:**
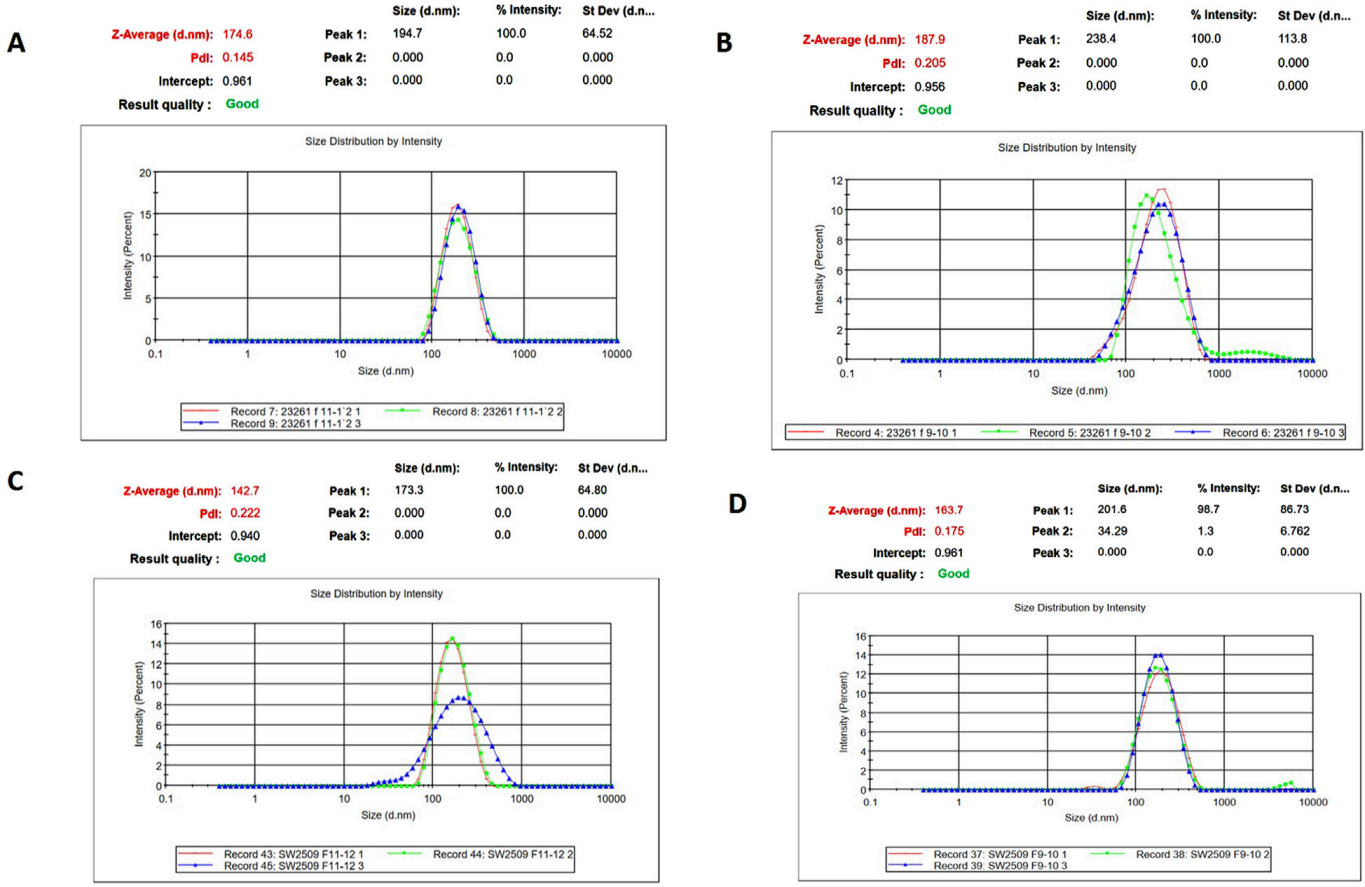
Characterization of seminal EVs. Intensity-based size distribution of SP-EVs analyzed by Zetasizer nano zs particle sizer. Each graph shows mean ± SD (n = 3), PDI, and particle size distribution for **(A)** Fractions 9-10 **(B)** fractions 11-12 of SP- EVs of HF bulls, and **(C)** fractions 9-10 **(D)** fractions 11-12 of seminal EVs from LF bulls.

**FIGURE 2:**
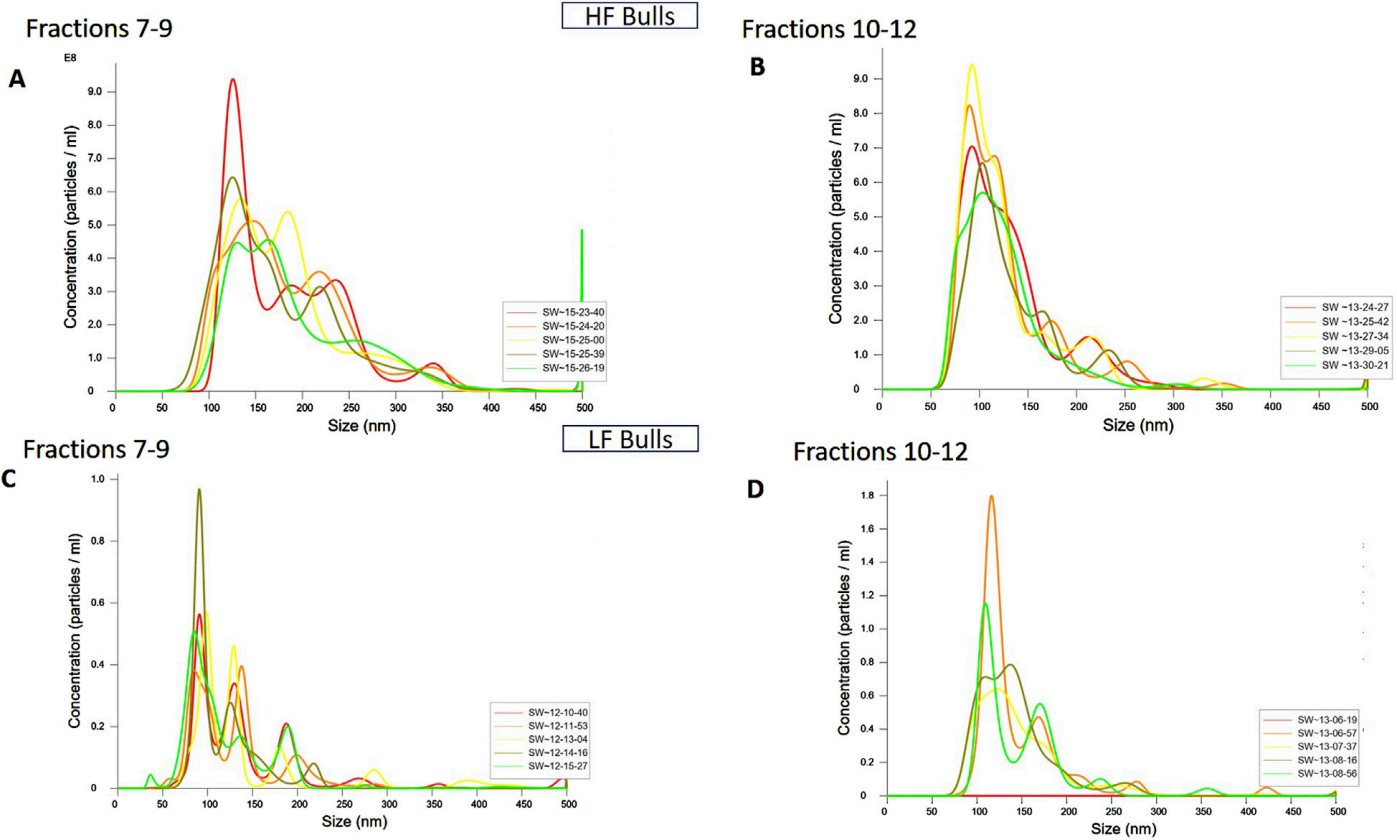
Characterization of seminal EVs Through Nanoparticle Tracking Analysis (NTA). The EVs were diluted 100X for NTA. The concentration vs. size graphs were plotted for **(A)** fractions 7-9, **(B)** 10-12 of HF bulls, and for fractions **(C)** 7-9, and **(D)** 10-12 of LF bulls.

**FIGURE 3:**
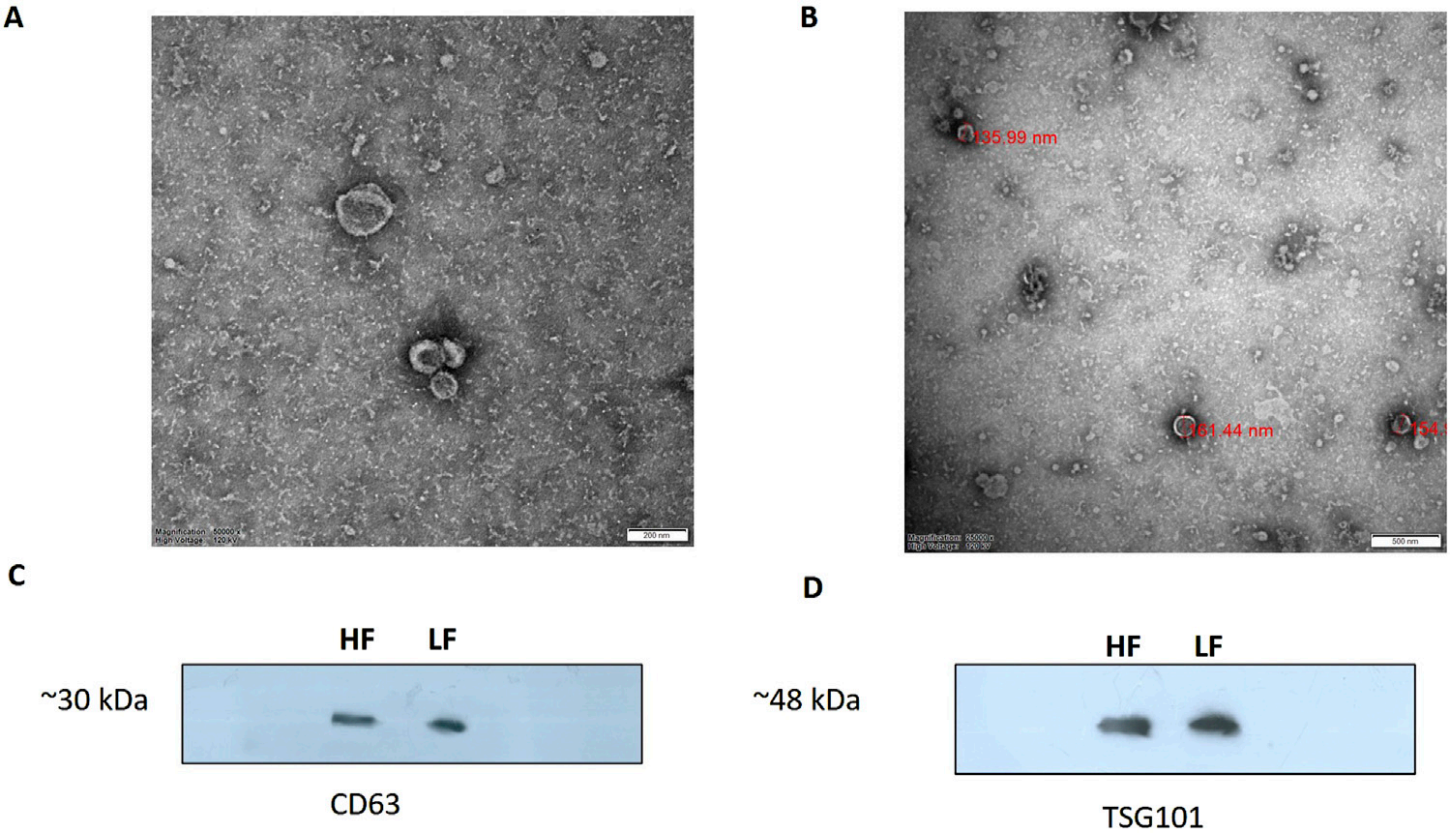
TEM image of EVs derived from seminal plasma **(A)** HF bulls **(B)** LF bulls SP-EVs. EVs were negatively stained with 1% phosphotungstic acid after removing the extra moisture (Magnification-250,000×, Scale bar-50 nm, 120 kV). Identification of the CD63 **(C)** and TSG101 **(D)** EV-specific protein markers by the western blot analysis of isolated pooled EVs samples from the seminal plasma of Sahiwal bull.

SP-EVs were isolated from high fertile (HF) and low fertile (LF) bull seminal plasma using size exclusion chromatography (SEC). The isolated SP-EVs fractions were characterized using differential light scattering (DLS), nanoparticle tracking assay (NTA), transmission electron microscopy (TEM), and Western blot. The size measurements through DLS (Figure 1) revealed that the SP-EVs of HF Sahiwal bulls ranged from 179.3 to 202 nm in fractions 7-8, 177–190 nm in fractions 9–10, 160–174 nm in fractions 11–12, and 178–190 nm in fractions 13–14. For LF bulls, the size range was found to be from 160 nm to 186 nm in fractions 7–8, 150 nm–180 nm in fractions 9–10, 142 nm–160 nm in fractions 11–12, and 140 nm–155 nm in fractions 13–14, in accordance with Böing et al. (2014) (Supplementary Tables S2, S3). The NTA results revealed that the SP-EV population size ranged, with a mean average particle size of 152.2 ± 1.8 nm in 10–12 fractions of HF bulls and 146.4 ± 1.1 nm in LF bulls. The concentration of SP-EVs through NTA was found to be 7.19 × 10^10^ to 7.23 × 10^10^ particles/mL in HF bulls and 4.23 × 10^10^ to 6.85 × 10^10^ particles/mL in LF bulls (Supplementary Tables S4, S5). The characterization using DLS and NTA revealed that SP-EVs were predominantly enriched in fractions 7-14, leading to discard the other fractions. Based on NTA analysis, neither the size nor the concentration were found to be significantly different between the HF and LF bull SP-EVs (Figure 2). The general morphology and ultrastructure of seminal plasma-derived EVs were examined using transmission electron microscopy (TEM), which revealed round, cup-shaped vesicles with lipid bilayer structures, ranging in diameter from 120 to 200 nm (Figures 3A, B). The presence of small EVs was further confirmed through Western blot analysis using EV protein markers, including CD63 and TSG101 (Figures 3C, D). Full blot films are shown in Supplementary Figure S2.

### miRNA profiling of SP-EVs of HF and LF bulls

**FIGURE 4:**
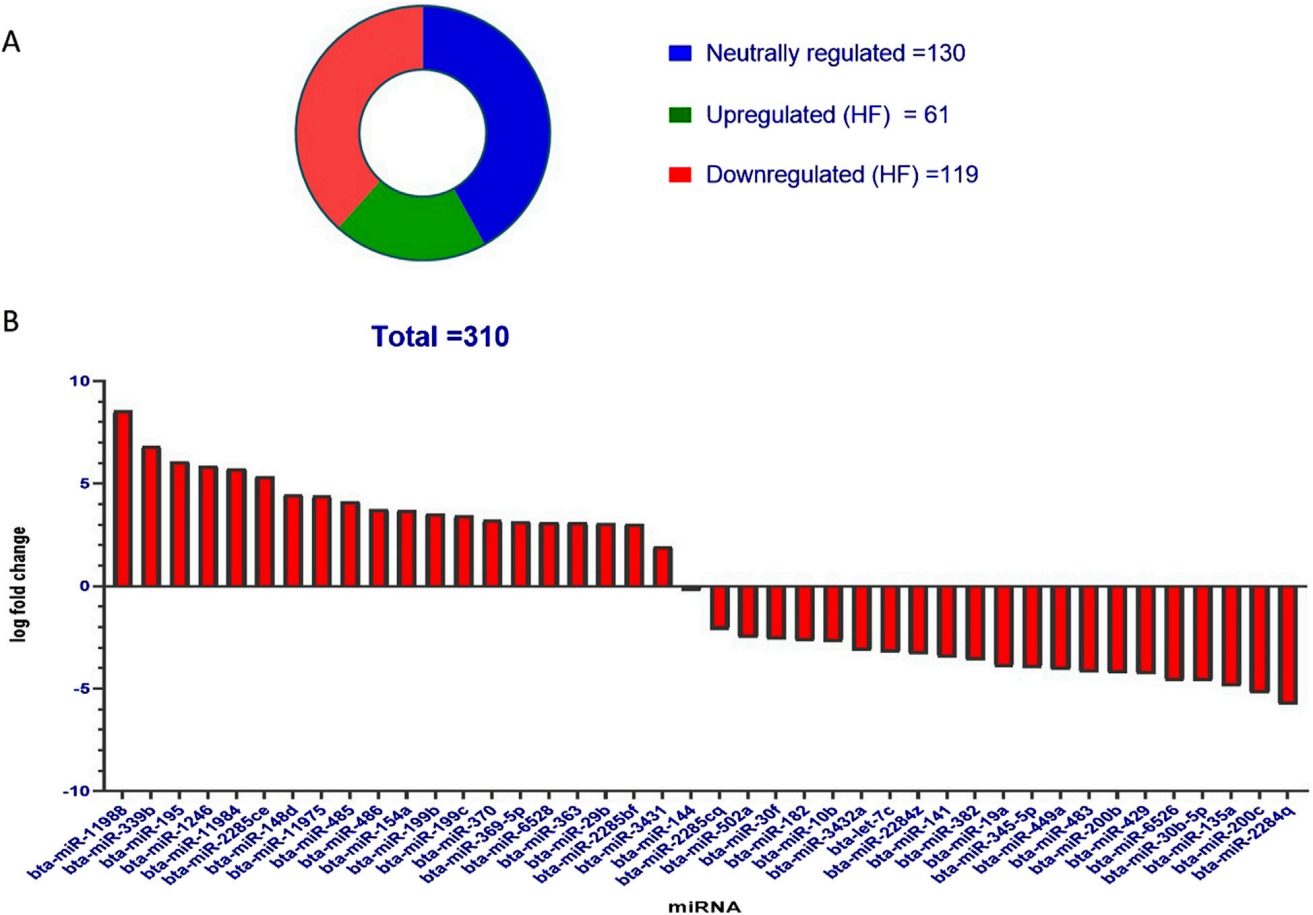
**(A)** Pie chart representation of the known miRNAs in SP-EVs. Total miRNAs identified in both HF and LF SP-EVs were 310 out of which 61 were upregulated in HF and 119 were downregulated in HF. **(B)** Bar graph showing significant upregulated and downregulated miRNAs in HF. The y-axis represents log fold change and the x-axis represents the shortlisted miRNAs in the decreasing order of their log fold changes.

DESeq normalized expression values were used to calculate the fold change for a given transcript based on the negative binomial distribution. In the case of HF samples, the transcripts that exhibited a log2-fold change less than −1 were represented as downregulated, while values greater than 1 were represented as upregulated, and values in between −1 and +1 were represented as neutrally regulated. A total of 310 miRNAs were fetched, out of which 61 were upregulated in HF, 119 were downregulated in HF, and 130 were neutrally regulated, i.e., there was no significant difference between the HF and LF groups (Figure 4A).

Out of the entire pool of 310 miRNAs, a more refined selection was made by considering both the p-value and the log-fold change. On the basis of a p-value threshold of less than 0.05 and a log fold change exceeding 1, a subset of 41 miRNAs were identified, of which 20 were upregulated in HF and 21 were downregulated in HF. The miRNAs exhibited upregulation in HF bulls, as indicated in Table 1 and Figure 4B.

**TABLE 1:** List of miRNAs that were upregulated in HF Sahiwal bulls.

	miRNA	p-value	Log-2 fold change
1	bta-miR-11988	2.62687E-08	8.549659501
2	bta-miR-339b	0	6.816110911
3	bta-miR-195	0.000225846	6.070151368
4	bta-miR-1246	0.010698653	5.870020001
5	bta-miR-11984	0.012182053	5.727158631
6	bta-miR-2285ce	0.0019668	5.345670832
7	bta-miR-148d	0.025819019	5.345670832
8	bta-miR-11975	0.0128763	4.468521855
9	bta-miR-485	0.033634156	4.418295281
10	bta-miR-486	0.021566833	4.139730938
11	bta-miR-154a	0.032283545	3.747902176
12	bta-miR-199b	0.032283545	3.728091214
13	bta-miR-199c	0.040928294	3.537478716
14	bta-miR-370	0.039906969	3.437638534
15	bta-miR-369-5p	0.040971771	3.164799435
16	bta-miR-6528	0.04779462	3.136983832
17	bta-miR-363	0.047461156	3.10301481
18	bta-miR-29b	0.024651537	3.082078833
19	bta-miR-2285b	0	3.037274482
20	bta-miR-3431	0	1.938069887

### *In-silico* target prediction of miRNA candidates

The target prediction of all the shortlisted 20 miRNAs that were upregulated in HF bulls was analyzed using online software such as Target Scan, miRwalk, miRmap, and Micro-T-CDS. Four miRNA candidates were selected, namely, bta-miR-339b, bta-miR-195, bta-miR1246, and bta-miR-199b. The clustering analysis of all predicted miRNA target genes is shown in Supplementary Tables S6–S9. Target genes identified by at least four databases were clustered using the Venn diagram (https://bioinformatics.psb.ugent.be/webtools/Venn/).

**FIGURE 5:**
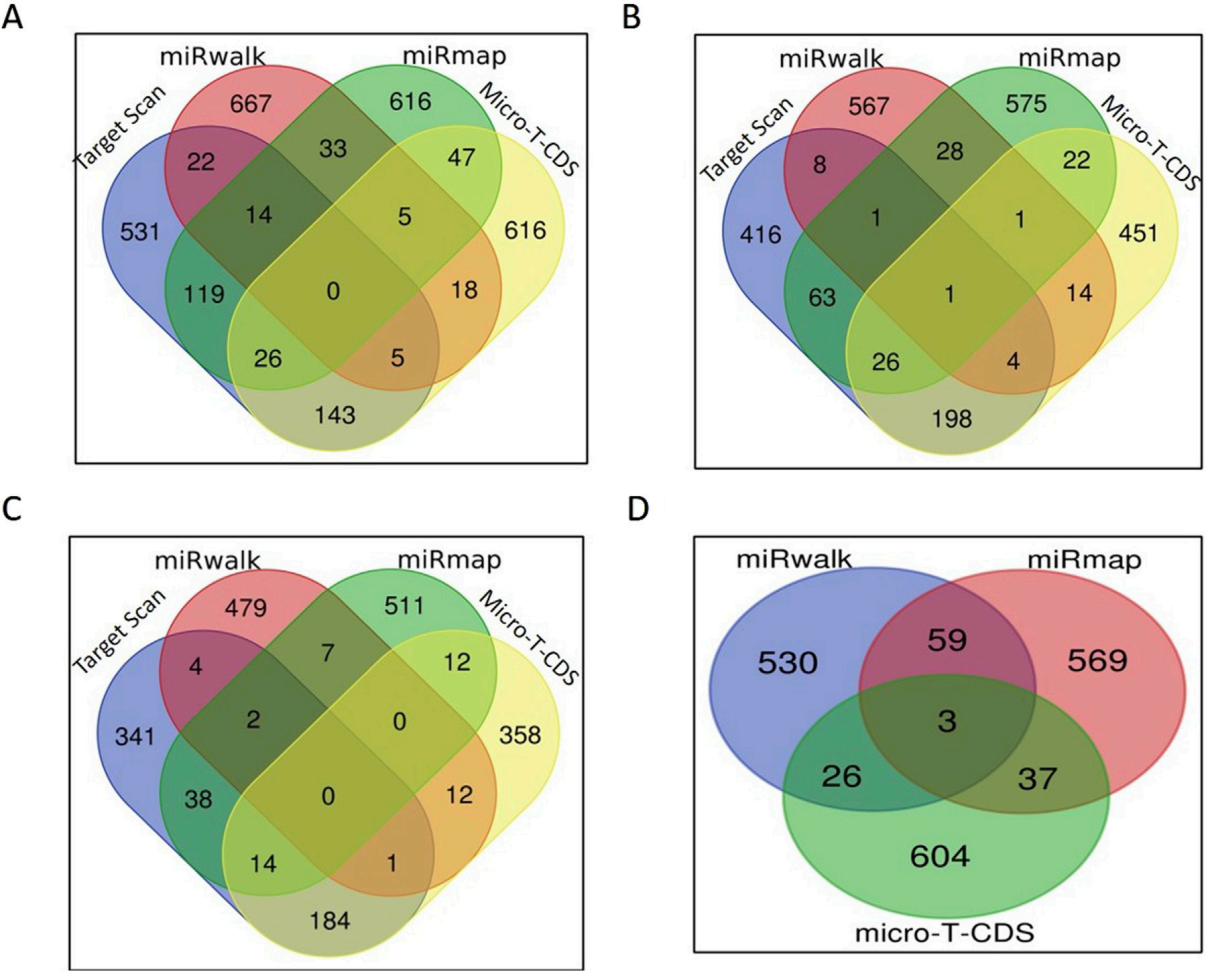
miRNA target prediction: **(A)** Venn diagram of the number of bta-miR-1246 targets predicted by each tool. **(B)** Venn diagram of the number of bta-miR-195 targets predicted by each tool. **(C)** Venn diagram of the number of bta-miR-199b targets predicted by each tool. **(D)** Venn diagram of the number of bta-miR-339b targets predicted by each tool.

For bta-miR-1246, when considering all four tools, the number of targets predicted by the intersection of TargetScan, miRwalk, and miRmap was 14; the intersection of TargetScan, miRwalk, and micro-T-CDS yielded five genes; the intersection of TargetScan, miRmap, and micro-T-CDS exhibited an overlap of 26 genes; and the intersection of miRmap, miRwalk, and micro-T-CDS showed five genes. Additionally, when considering pairs of target prediction tools, TargetScan and miRwalk had 22 common genes, whereas 119 genes were common between TargetScan and miRmap. The overlap between TargetScan and micro-T-CDS was even more significant, with 143 common genes. Moreover, miRmap and miRwalk displayed an overlap of 33 genes, while miRwalk and micro-T-CDS had 18 genes in common. Finally, the tools micro-T-CDS and miRmap shared 47 genes (Figure 5A).

In the case of bta-miR-195, one gene was common among all four tools. While considering the overlap of three softwares, four genes were found to be common in TargetScan, miRwalk, and micro-T-CDS, whereas 26 genes were found common in TargetScan, miRmap, and micro-T-CDS. An overlap of only one gene was observed between miRmap, miRwalk, and micro-T-CDS, and eight genes were identified as common in both TargetScan and miRwalk. A total of 63 genes were found common between TargetScan and miRmap; 198 genes overlapped in TargetScan and micro-T-CDS; and 28 genes were identified as common in miRmap and miRwalk. Among miRwalk and micro-T-CDS, 14 genes showed overlap, and lastly, 22 genes were found common between miRmap and micro-T-CDS (Figure 5B).

In the case of bta-miR-199b, none of the genes were found to be common among all four softwares. Two genes were common in TargetScan, miRmap, and miRwalk, while one gene was common in TargetScan, miRwalk, and micro-T-CDS. Fourteen genes were found to be common in TargetScan, miRmap, and micro-T-CDS. When considering two tools, our genes overlapped between TargetScan and miRwalk, while 38 genes were shared between TargetScan and miRmap. A total of 184 genes were found to be common in TargetScan and micro-T-CDS, and seven genes were found to overlap between miRmap and miRwalk. Among miRwalk and micro-T-CDS, twelve genes were found to be shared, while two genes were found to be common between miRmap and micro-T-CDS (Figure 5C).

In the case of bta-miR-339b, target prediction was done through miRmap, miRwalk, and micro-T-CDS (Figure 4). Due to the small number of gene candidates in the software Target Scan, it was not employed for this miRNA. Three genes were found to overlap among them, while 59 genes were common among miRmap and miRwalk. A total of 26 genes were found to be shared between miRwalk and micro-T-CDS (Figure 5D).

### Gene ontology and functional pathway enrichment analysis for bta-miR-1246 targeted genes

**FIGURE 6:**
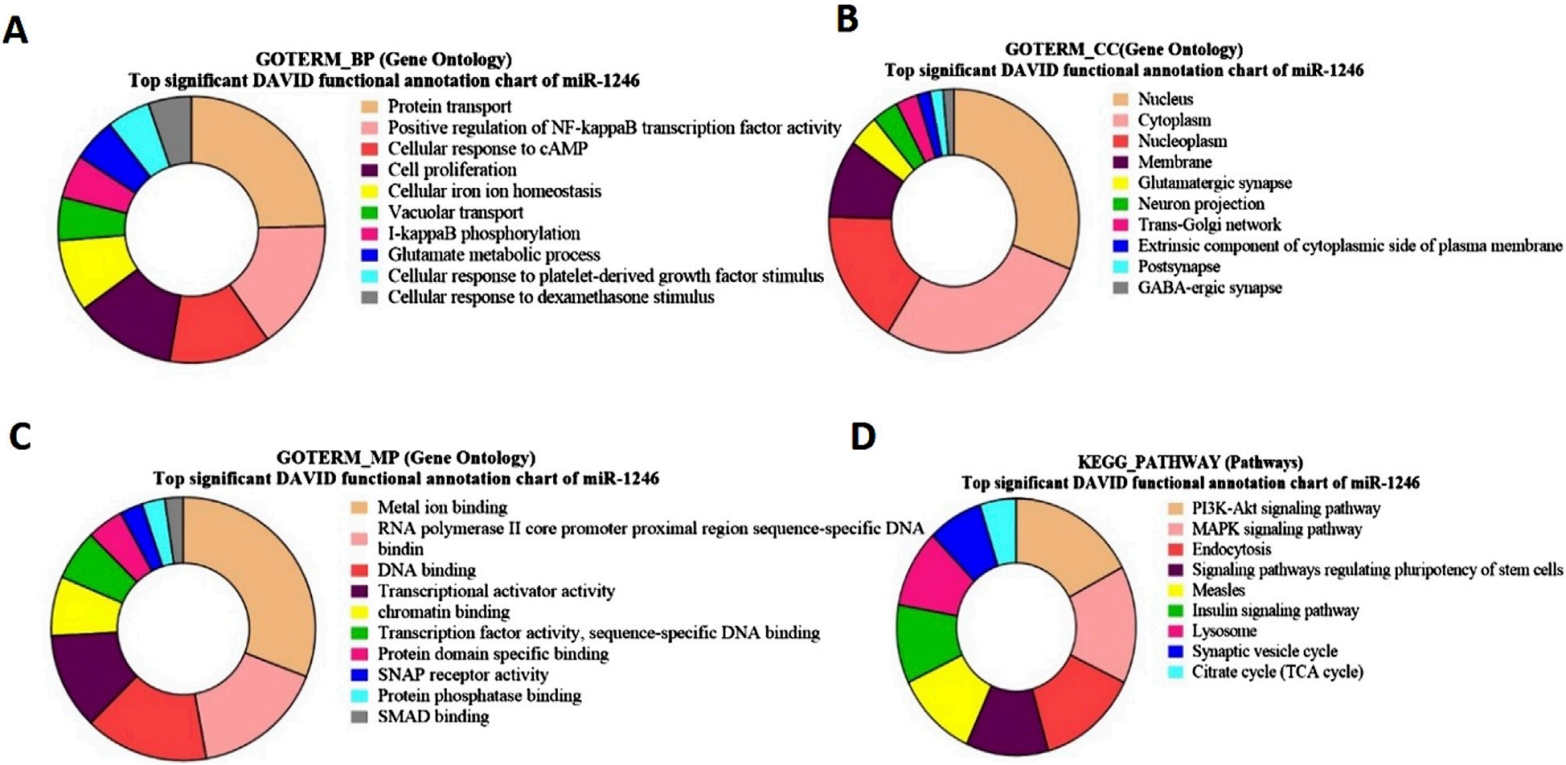
Pie charts representing top Gene Ontology (GO) terms and pathway analysis for bta-miR-1246 with respect to **(A)** Biological Process (BP), **(B)** Cellular Component (CC) **(C)** Molecular Functions (MF) and **(D)** KEGG Pathway.

For bta-miR-1246 target genes, the Gene Ontology (GO) term biological process revealed a significant involvement of genes in protein transport (Figure 6A). Notably, some of the identified genes, such as CFTR, ARL3b, and AXDND1 (*p* = 1.5E-4), have been involved in spermiogenesis and in the differentiation of spermatids into mature sperm Regarding the GO term analysis of molecular function for bta-miR-1246, a notable enrichment was observed for metal ion binding such as SMAD4, (Figure 6C). The GO term cellular components revealed a significant enrichment of genes in the nucleus and the cytoplasm such as GMCL1 (p = 6.92E-06), and PICK1 (p = 2.2E-6), has been found to be associated with spermatogenesis (Figure 6B).

The KEGG pathway of bta-miR-1246 target genes such as FGF7 and GHR (p = 5.7E-4), revealed significant associations with the PI3-Akt pathway and the MAPK signaling pathway (Figure 6D; Supplementary Table S10).

### GO and KEGG pathway enrichment analysis for bta-miR-195 targeted genes

**FIGURE 7:**
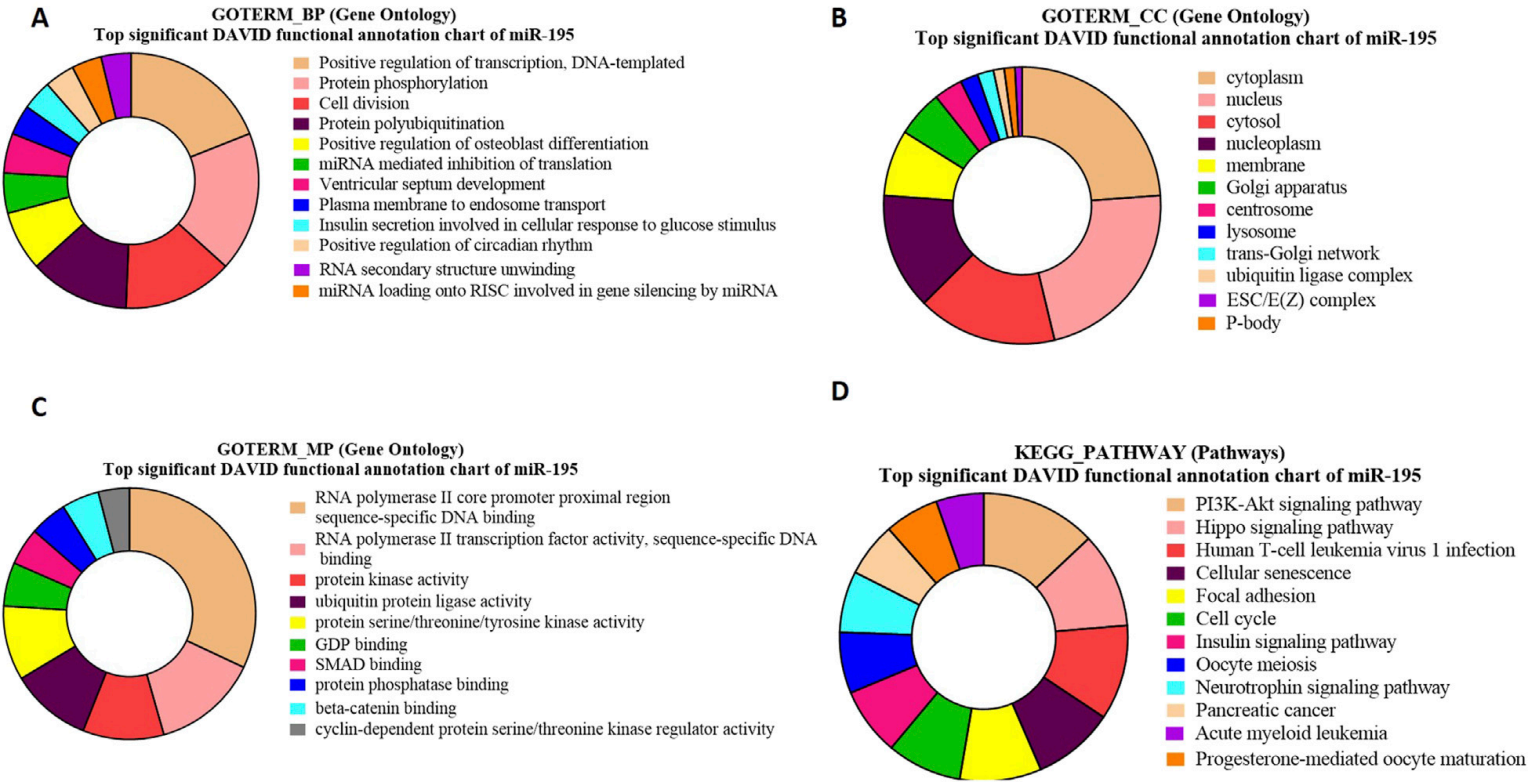
Pie charts representing top 10 Gene Ontology (GO) terms and pathway analysis for bta-miR-195 concerning **(A)** Biological Process (BP), **(B)** Cellular Component (CC) **(C)** Molecular Functions (MF) and **(D)** KEGG Pathway.

The GO term biological processes in the case of bta-miR-195 target genes revealed a significant enrichment of these genes (PIM1, RAF, HMGA1, p = 2.3E-5) in positive regulation of transcription, DNA templated (p = 1.89E-04) (Figure 7A).

The GO term cellular components for bta-miR-195 target genes revealed that the maximum number of genes were present in the cytoplasm (ARL2, ARL3, BART, p = 1.4E-13) (Figure 7B). The GO term molecular functions revealed that the maximum number of genes were associated with several important functions, including (NDP, FBXL20 *p* = 1.7E-4), the sequence-specific SMAD binding, and protein kinase activity (Figure 7C).

The KEGG pathway enrichment analysis of bta-miR-195 target genes revealed that the maximum number of genes (LCRMP1, AKT3, *p* = 9.0E-6) were associated with the PI3K-Akt-signaling pathway (Figure 7D). Some of the identified genes of these pathways, such as. AKT3, play a crucial role in the proliferation of precursor cells in spermatogenesis, which needs tight regulation in order to maintain a normal sperm count without any abnormal spermatozoa (Wang et al., 2021). LCRMP 1 (p = 9.66E-06) is known to play crucial roles in the PI3-Akt and Ras signaling pathways. This gene is abundantly present in the testis, and upon its knockout in mice, it exhibits aberrant spermiation with apoptotic spermatids, suggesting its important role in spermatogenesis (Chang et al., 2023) (Supplementarty Table S11).

### GO and KEGG pathway enrichment analysis for bta-miR-399b-targeted genes

**FIGURE 8:**
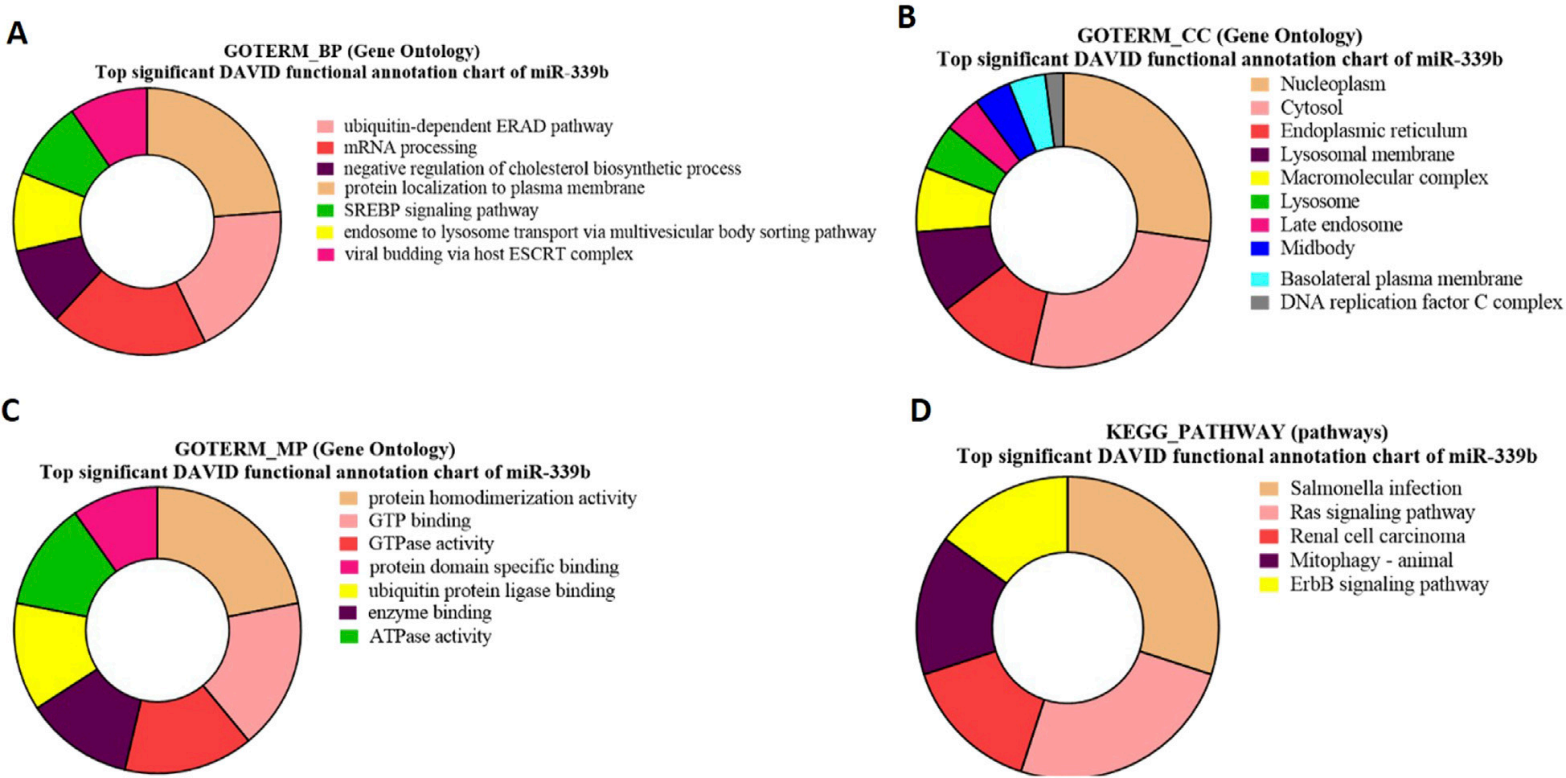
Pie charts representing top 10 Gene Ontology (GO) terms and pathway analysis for bta-miR-339b with respect to **(A)** Biological Process (BP), **(B)** Cellular Component (CC) **(C)** Molecular Functions (MF) and **(D)** KEGG Pathway.

GO-term biological processes for bta-miR-339b target genes revealed significant enrichment of these genes associated with the ubiquitin-dependent ERAD pathway (Figure 8A). The gene TSPAN1 (p 0.012549) and p53 (p = 0.03655) identified in GO biological process, which is known to be an EV biomarker, therefore suggesting a link between bta-miR-339b and EV production and The gene p53 (p = 0.03655) is involved in the apoptosis of abnormal sperm.

The GO term molecular process for bta-miR-339b target genes revealed that a maximum number of genes were associated with protein homodimerization activity and indicated that bta-miR-339b is associated with specific genes, including CCNB2 (p = 0.008349) (Figure 8C).

Gene Ontology (GO) term cellular components showed enrichment of bta-miR-339b target genes within the nucleoplasm such as ALDP (p = 1.09E-04) and ZDHHC19 (p = 1.09E-04) (Figure 8B).

The functional analysis of bta-miR-339b target genes using KEGG pathway enrichment led to the identification of genes, i.e (p = 0.049008358), associated with the Ras signaling pathway (Figure 8D). The identification of the RABL2 target of bta-miR-339b highlights the potential regulatory role of this miRNA in signal transduction and during spermatogenesis (Supplementary Table S12).

### Gene ontology and KEGG pathway enrichment analysis for bta-miR-199b-targeted genes

**FIGURE 9:**
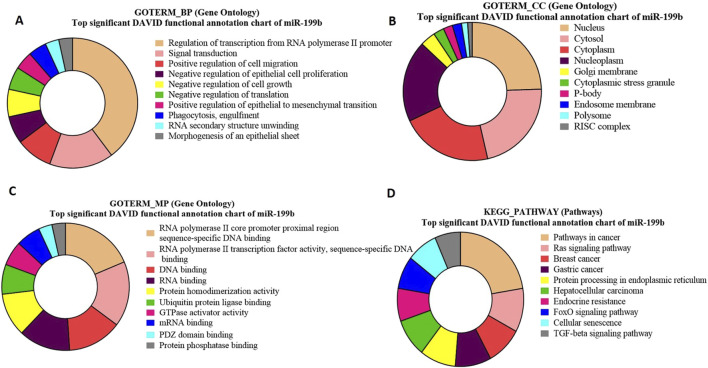
Pie charts representing top 10 Gene Ontology (GO) terms and pathway analysis for bta-miR-199b concerning **(A)** Biological Process (BP), **(B)** Cellular Component (CC) **(C)** Molecular Functions (MF) and **(D)** KEGG Pathway.

GO analysis of bta-miR-199b target genes highlights a significant enrichment of genes associated with crucial biological processes (Figure 9A), viz., regulation of transcription and signal transduction. Moreover, the functional analysis identified the genes SMAD2 and DAZ (p = 0.010716) as significant targets of bta-miR-199b. The GO term molecular function for bta-miR-199b revealed that the maximum number of genes were associated with RNA Polymerase II Core Promoter Proximal Region Sequence-Specific DNA Binding, GTPase Activity, and RNA Binding (Figure 9C), which play roles in transcriptional regulation as well as cell signaling in the cells.

The GO term cellular components for bta-miR-199b targets revealed that these are predominantly located in the nucleus Among the genes located in the nucleus, FXR1 was identified to be of particular significance (p = 6.78E-04) (Figure 9B).

The functional analysis of bta-miR-199b using the KEGG pathway analysis revealed that the Ras signaling pathway is enriched with the maximum number of genes associated with this miRNA. Within the Ras Signaling Pathway (Figure 9D), specific genes, such as cKIT and RB1 (p = 0.035763), were identified as significant targets of bta-miR-199b.

### Relative expression analysis of selected miRNAs between HF and LF bull spermatozoa

**FIGURE 10:**
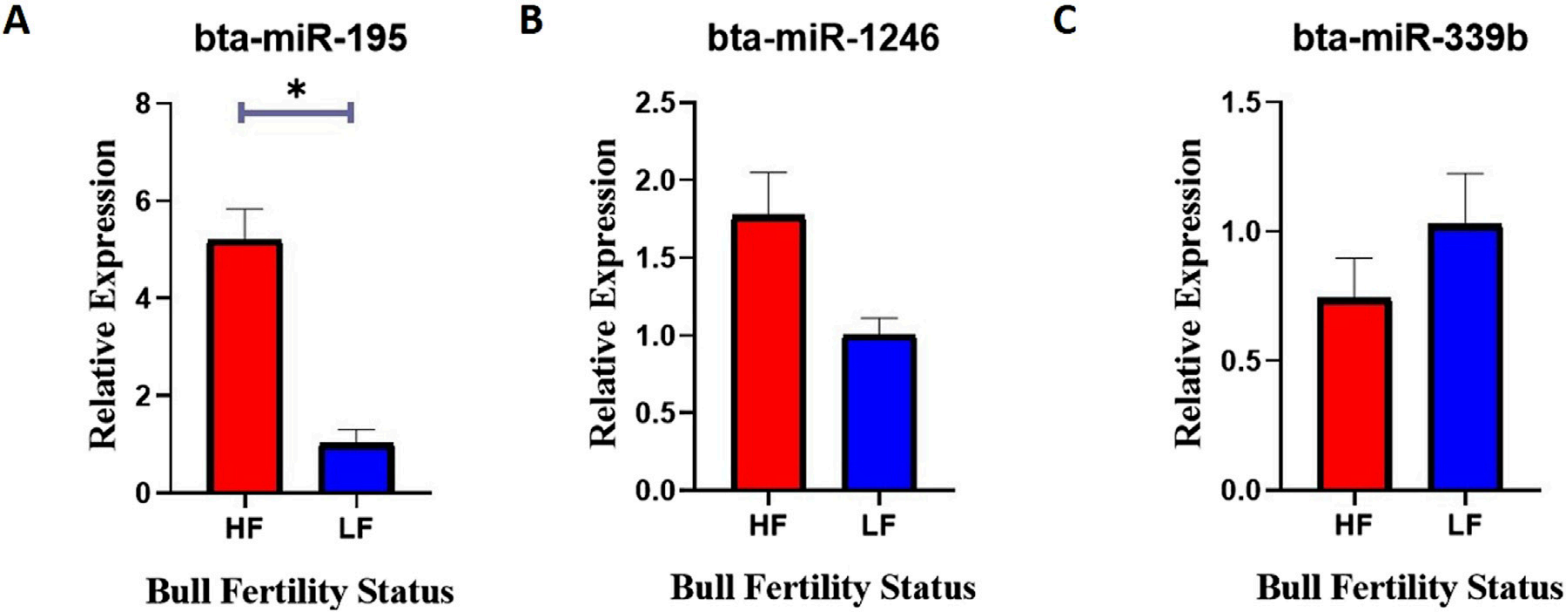
Relative expression of **(A)** bta-miR-195, **(B)** bta-miR-1246, and **(C)** bta-miR-339b between the spermatozoa of HF and LF bulls. Expression values were normalized to let-7a miRNA. The -axis represents the bull fertility status and the y-axis represents the fold change. Vertical bars represent means of fold change and error bars represent the standard error of mean at p = 0.05.

Semen samples were collected from HF and LF bulls, and miRNA was isolated from the sperm cells using the miRneasy kit. RT-qPCR was performed to measure the relative expression levels of specific miRNAs in HF and LF bulls. The expression of bta-miR-195 was found to be significantly 80% higher (p-value <0.05) in HF bulls compared to LF bulls (Figure 10A). This indicates that bta-miR-195 is strongly associated with the uptake of bta-miR-195 on sperm, may play a role in regulating processes related to fertility, and could be associated with improved reproductive outcomes in HF bulls. However, no significant differences in expression were observed for bta-miR-1246 and bta-miR-339b between HF and LF bulls (Figures 10B, C). Though both miRNAs were differentially different in both HF and LF bulls. This suggests that these miRNAs may not be directly linked to fertility-related processes in the context of Sahiwal bulls. Unfortunately, due to a primer error, the amplification of bta-miR-199b could not be achieved and, hence, was excluded from this study.

Mounting evidence from this study suggests that the high expression of bta-miR-195 appears to have a potential association with the fertility status of Sahiwal bulls. It can be supported that the gene ontology and KEGG pathway analysis previously discussed have shown that overexpression of certain bta-miR-195 target genes, such as PIM1, AKT3, and FBXL20, could have detrimental effects on spermatozoa and the spermatogenesis process. As a result, it is hypothesized that bta-miR-195 might positively impact fertility in HF bulls by down-regulating the above genes.

## Discussion

The unpredictability and limitations in current semen evaluation techniques in the assessment of bull fertility more precisely led us to explore the SP-EVs transcript-based alternative methods for predicting Sahiwal bull fertility. In this study, we hypothesize that the presence of miRNAs in SP-EVs plays a crucial role in regulating the fertility of Sahiwal bulls. In addition, the differential expression of miRNAs in SP-EVs and their role in fertility remain unexplained in indigenous cattle. So, this investigation was specifically designed to determine how the variations in the abundance of specific miRNAs in SP-EVs are related with Sahiwal bull fertility.

Herein, SP-EVs were isolated using SEC and characterized by DLS, NTA, TEM, and Western blotting. Fractions 7–14 contained EVs with a mean size of 200 nm in both fertility groups, consistent with findings from previous studies (Barranco et al., 2023). The particles in the sample were homogeneous, as indicated by a polydispersity index (PDI) ranging from 0.2 to 0.35 in the DLS data from EVs of both fertility groups. Additionally, the NTA assay not only confirmed the size of the seminal EVs but also verified their abundance in cattle seminal plasma. TEM also revealed the characteristic round, cup-shaped morphology of seminal EVs in both HF and LF bulls, within the desired size range (Xu et al., 2020). miRNA profiling of SP-EVs from high-fertility (HF) and low-fertility (LF) Sahiwal bulls was performed using high-throughput RNA sequencing. The present study identified a pool of 310 miRNAs. By refining the selection based on a p-value (< 0.05) and a log-fold change greater than one, a subset of 41 miRNAs were identified. Of these, 20 miRNAs were upregulated in high-fertility (HF) bulls and 21 were downregulated in HF bulls (Table 1), respectively. Our results are in concordance with the earlier studies by Dlamini et al. (2023).

The microRNAs have the ability to control target genes at the post-transcriptional level by either repressing their translation or causing the degradation of nucleic acids, thereby inhibiting gene expression (O’Brien et al., 2018). In accordance with previous literature, certain X-linked miRNAs are believed to play a crucial role in spermatogenesis and male reproductive processes (Li et al., 2014; Qing et al., 2017). Among the most abundant miRNAs, several were common across datasets, such as bta-miR-195 (Do et al., 2019), bta-miR-1246 (Salas-Huetos and Aston, 2021), bta-miR-486 (Donnellan et al., 2022), and bta-miR-199b (Kasimanickam et al., 2022). These miRNAs are anticipated to play crucial regulatory roles in diverse physiological processes within sperm. Additionally, gene targets for the identified miRNAs were predicted using various online tools (Pal et al., 2023). We selected four miRNAs—bta-miR-1246, bta-miR-195, bta-miR-339b, and bta-miR-199b—because they had the highest number of predicted target genes. As it is well known that, extracellular vesicles (EVs) play a crucial role in transmitting signals involved in cellular functions, including the maturation of germ cells, and EVs can transfer a variety of RNA molecules to sperm (Ding et al., 2021), Next we envisaged to uncover critical insights into the influence of miRNAs on sperm function and fertility by conducting gene ontology and KEGG pathway analyses.

The GO term biological process for bta-miR-1246 indicated a significant involvement of genes such as ARL3b and AXDND1 both of which are recognized for their critical roles in spermiogenesis and the differentiation of spermatids into mature sperm. These genes are particularly integral to protein transport pathways, suggesting that bta-miR-1246 may play a regulatory role in these essential reproductive processes (Ma et al., 2021; Qi et al., 2013). GO term analysis of bta-miR-1246 also revealed significant enrichment for metal ion binding. SMAD4, a metal ion-binding protein, mediates TGF-β signaling and is crucial for testicular development. Overexpression of SMAD4 has been linked to spermatogenic arrest and seminiferous tubule degradation (Narula et al., 2002). Our findings suggest a positive correlation, as bta-miR-1246 is upregulated in high fertility (HF), potentially regulating SMAD4 expression to facilitate normal spermatogenesis. GO-term analysis of cellular components revealed significant enrichment of genes in the nucleus and cytoplasm, including GMCL1, which is essential for nuclear envelope formation and spermatogenesis. GMCL1 knockout leads to abnormal or absent sperm production (Azhar et al., 2021). Another gene, PICK1 (p = 2.2E-6), is associated with spermatogenesis, and its downregulation has been linked to disruptions in the acrosome reaction (Du et al., 2023).

The pathway analysis of target genes regulated by bta-miR-1246 revealed significant associations with the PI3-Akt pathway and the MAPK signaling pathway. Genes involved, FGF and GHR, play roles in testicular maturation and contribute to germ cell production by releasing fibroblast growth factors and testosterone, crucial for testicle development. These genes also support sperm motility improvement (Garbarino Azúa et al., 2017; Jiang et al., 2013). Therefore, bta-miR-1246 miRNA is also implicated in the regulation of sperm motility Biological process of bta-miR-195 target genes showed significant enrichment in the positive regulation of transcription, DNA-templated processes. Genes such as HMGA1 and PIM1 are involved in spermatocyte-to-spermatid differentiation, with PIM1 upregulation potentially causing harmful inflammation to spermatozoa (Jiménez-Garciá et al., 2016). In cellular components for bta-miR-195 target genes, the majority of genes were found in the cytoplasm. ARL2 and BART are typically cytosolic but can translocate to mitochondria, where they bind to adenine nucleotide transporters, potentially influencing energy metabolism during spermatogenesis (Sharer et al., 2002). In the molecular function, a significant number of genes were associated with various important functions, including RNA polymerase activity, sequence-specific DNA binding, and protein homodimerization. Among these genes, NDPencodes nucleoside diphosphate (NDP) kinases that are crucial during spermiogenesis. Another identified gene, FBXL20, plays a role in inflammatory responses which can be detrimental to spermatozoa and ultimately lead to infertility (Yang et al., 2019). The pathway enrichment analysis of bta-miR-195 target genes revealed that the maximum number of genes were associated with the Ras signaling pathway.

The gene TSPAN1, identified in the GO biological process, encodes a tetraspanin protein known to serve as an extracellular vesicle (EV) biomarker, implying a potential association between bta-miR-339b and EV production (Jankovičová et al., 2020). The enrichment of TSPAN1 and p53 indicates that bta-miR-339b likely plays a critical role in regulating this pathway, which is essential for maintaining protein homeostasis and cellular integrity. In the molecular processes for bta-miR-339b target genes, a significant number of genes were associated with protein homodimerization activity. Specifically, bta-miR-339b was found to regulate genes such as CCNB2 (p = 8.5E-3), a member of the cyclin family critical for G2/M cell cycle progression through activation of CDK1. CCNB2 is expressed in testicular germ cells and is essential for spermatogenesis (Shen et al., 2022).

The cellular components revealed the enrichment of bta-miR-339b target genes within the nucleoplasm. Of particular interest, specific genes such as ALDP (p = 1.09E-04) are involved in the degradation process of very long-chain fatty acids during spermatogenesis (Linn et al., 2021). ZDHHC19 (p = 1.09E-04) is another gene identified as crucial for normal sperm function; its absence in spermatozoa leads to multiple defects, including abnormal sperm tail morphology, reduced motility, and disrupted acrosome reactions (Wang et al., 2021). The pathway functional analysis of bta-miR-339b target genes led to the identification of genes associated with the Ras signaling pathway. In the Ras signaling pathway, RABL2 was identified as significantly associated, playing a crucial role in signal transduction as a key mediator during spermatogenesis (Lo et al., 2012).

Furthermore, In the biological processes of bta-miR-199b target genes (SMAD2 and DAZ) are involved in transcription regulation and signal transduction. SMAD2 is present in testis, sertoli cells, and preleptotene to pachytene spermatocytes but not in post-meiotic germ cells, suggesting its involvement in spermatocyte differentiation (Wang and Zhao, 1999). DAZ, a highly conserved RNA-binding protein, is essential for gametogenesis in metazoans. It plays a role in regulating the translation of specific mRNAs, and its absence halts the process of spermatogenesis (Reynolds and Cooke, 2005). The GO analysis of cellular components for bta-miR-199b targets showed predominant localization in the nucleus. Notably, FXR1 emerged as significant among these nuclear genes. Downregulation or knockout of FXR1 has been associated with disrupted spermiogenesis, which is implicated in infertility (Kang et al., 2022).

In addition, the pathway functional analysis revealed that the Ras signaling pathway is enriched with the maximum number of genes associated with this miRNA. Specific genes, such as cKIT and RB1, were identified as significant targets of bta-miR-199b. The cKIT regulates primordial germ cell migration, proliferation, and apoptosis during fetal gonadal development (Mauduit et al., 1999). RB1 (Retinoblastoma 1) is a tumor suppressor gene known to regulate cell cycle progression. In the context of spermatogenesis, RB1 plays a critical role in controlling meiotic cell division, ensuring the proper development of haploid spermatocytes and oocytes, and maintaining the spermatogonial stem cell pool (Yang et al., 2019). The gene ontology and pathway analysis of the target genes for the four selected miRNAs, which are abundant in SP-EVs, indicate that many of these genes are associated with functions that either positively or negatively impact spermatogenesis and reproductive functions.

Following the functional analysis of various miRNAs associated with fertility, we proceeded to compare the relative expression of selected miRNAs between the spermatozoa of high-fertility (HF) and low-fertility (LF) bulls. The significant increase in the detection of bta-miR-195 in HF spermatozoa indicates that bta-miR-195 is closely associated with sperm uptake and may play a regulatory role in fertility-related processes, potentially contributing to improved reproductive outcomes in HF bulls. However, no significant differences in expression were observed for bta-miR-1246 and bta-miR-339b between HF and LF bulls, despite their differential expression patterns in both groups, highlighting that in HF spermatozoa these miRNAs abundance did not perfectly relate with bull fertility.

The findings of this study suggest a potential link between the high expression of bta-miR-195 indicating its capacity to regulate the target genes, such as PIM1, AKT3, and FBXL20 may negatively impact spermatozoa and the spermatogenesis process in highly fertile bulls with superior conception rates.

Additionally, due to logistical and financial limitations, we were able to collect semen samples from the bulls only twice a week, as the study relied entirely on standing bulls. This limited the availability of samples. For the purposes of characterization in the western blot experiment (for protein isolation) and sequencing (RNA isolation), we pooled fractions of SP-EVs. Pooling samples reduces the sample size while maintaining a high degree of confidence in the data (Somashekar et al., 2017). It increases the likelihood of detecting the maximum number of miRNAs with minimal inter-individual variation within a specific treatment group. Further experiments involving data analysis with a higher number of bulls within a specific (HF and LF) group will certainly help in gaining deeper and more robust insights into the findings of the current study.

## Conclusion

In this study, we developed the miRNA profile of SP-EVs in contrasting fertility Sahiwal bulls using high-throughput RNA sequencing. Our findings suggest that the fertility status of Sahiwal bulls is not influenced by the size and concentration of SP-EVs. However, the miRNA profile of SP-EVs reveals numerous differentially abundant miRNAs. Based on target prediction of these miRNAs, followed by gene ontology and pathway analysis, indicate that the target of bta-miR-195 is associated with various sperm functions, such as sperm motility and acrosome reaction. Additionally, while examining the expression of these miRNAs in spermatozoa, bta-miR-195 exhibited approximately 80% higher expression in high-fertility bulls compared to the low-fertility group (p < 0.05), Highlighting a potential association between bta-miR-195 and the fertility status of Sahiwal bulls. Taken together leads clearly suggest that bta-miR-195 promises as a potential fertility indicator in Sahiwal bulls. Further validation of differentially abundant miRNAs as well as assessment of their transcripts in distinct fertility bulls could significantly enhance our understanding of miRNA in male fertility in Sahiwal cattle bulls.

## Data Availability

The datasets presented in this study are included within the manuscript and its Supplementary Material. Raw data supporting the findings of this study are openly available in an online repository. The name(s) of the repository/repositories and the corresponding details can be found below: https://www.ncbi.nlm.nih.gov/sra/PRJNA1147473, accession number PRJNA1147473.
