# Supplementary material for: Differential abundance of microRNAs in seminal plasma extracellular vesicles (EVs) in Sahiwal cattle bull related to male fertility
Source: Front Cell Dev Biol. 2024 Oct 1;12:1473825. doi: 10.3389/fcell.2024.1473825 (PMC11473417; doi:10.3389/fcell.2024.1473825)
Supplement: Supplementary file 1 [file DataSheet1.docx]

Supplementary Data

Supplementary Table S1: Selected bulls and their conception rates (CR).

| **Fertility groups** | **Total Al** | **Pregnancy** | **Conception Rate (%)** |
| --- | --- | --- | --- |
| HF1 | 99 | 59 | 59.59 |
| HF2 | 54 | 32 | 59.25 |
| HF3 | 43 | 24 | 55.81 |
| LF1 | 199 | 58 | 29.15 |
| LF2 | 78 | 21 | 26.15 |
| LF3 | 23 | 7 | 30.43 |

Supplementary Data Figure S1: Selected bulls and their conception rates (CR).

The normal distribution of CR of 32 bulls. Six Cow bulls n=6, CR =55.81 – 59.59 % HF n=3, CR= 29.15 – 30.43% are LF (n=3) chosen for the study had their CR above and below the Mean ±1 SD (HF >52.6% and LF< 36.6%)

Supplementary Table S2: SP-EVs size distribution (nm) of fraction 7-14 in HF Bulls

| HF Bull | F7-8 | F9-10 | F11-12 | F13-14 |
| --- | --- | --- | --- | --- |
| HF 1 | 201.6 | 187.9 | 174.6 | 190 |
| HF 2 | 192.1 | 161.9 | 160 | 145.4 |
| HF 3 | 179.3 | 177.5 | 160 | 178 |

Supplementary Table S3: SP-EVs size distribution (nm) of fraction 7-14 in LF Bulls (n=3)

| LF Bull No | F7-8 | F9-10 | F11-12 | F13-14 |
| --- | --- | --- | --- | --- |
| LF 1 | 186.7 | 176 | 162.6 | 155.6 |
| LF 2 | 168.5 | 160 | 142.7 | 140.0 |
| LF 3 | 163.5 | 152.4 | 160 | 141.3 |

Supplementary Table S4: Concentration (particles/ml) of SP-EVs in different fractions of HF bulls (n=3)

| HF | F 7-9 | F 10-12 | F 13-15 |
| --- | --- | --- | --- |
| HF r1 | 7.16 × 10^10^ | 7.23 × 10^10^ | 5.76 × 10^10^ |
| HF r2 | 7.25 × 10^10^ | 7.28 × 10^10^ | 5.31 × 10^10^ |
| HF r3 | 6.59 × 10^10^ | 7.19 × 10^10^ | 5.86 × 10^10^ |

Supplementary Table S5: Concentration (particles/ml) of SP-EVs in different fractions of LF bulls (n=3)

| LF | F 7-9 | F 10-12 | F 13-15 |
| --- | --- | --- | --- |
| LF r1 | 2.66 × 10^11^ | 6.24 × 10^10^ | 6.24 × 10^10^ |
| LF r2 | 2.31 × 10^11^ | 6.68 × 10^10^ | 6.40 × 10^10^ |
| LF r3 | 2.70 × 10^11^ | 6.58 × 10^10^ | 5.30× 10^10^ |

Supplementary data – Figure S2: Western blot results of CD63 and TSG101


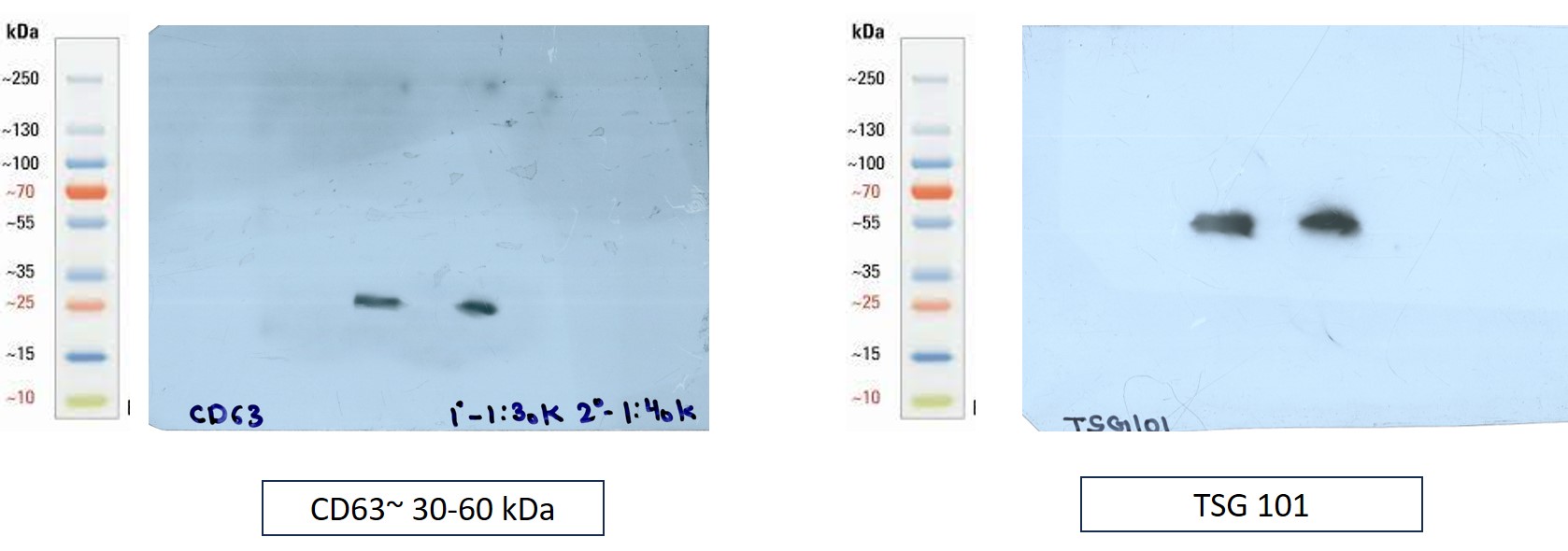


Supplementary Table S6: List of miRNA target predicted by various tools for miRNA (bta-miR-195)

| Name of Target prediction tool | Targets count | Genes |
| --- | --- | --- |
| Target Scan, miRmap, ,miRwalk micro-T-CDS | 1 | NUP50 |
| Target Scan miRmap miRwalk | 1 | CAPZA2 |
| Target Scan miRwalk micro-T-CDS | 4 | \| HTR2A, KDSR, DCAF7, MTMR 11 \| \| --- \| |
| Target Scan miRmap micro-T-CDS | 26 | KCNJ2, RBM6, IRAK2, ATXN7L3, MAPK8, ZNF423, SEMA6D, RNF24, PPP6C, SH3GL2, OMG, NDP. XPO7, KANK1, NAPG, PPAP2A, RAB11FIP2, BCL2L2, NRBP1, PPP2R5C, TBP, SEH1L, CREBL2, JARID2, RNF125, GLS2   \|  \| \| --- \| \|  \| |
| miRmap miRwalk micro-T-CDS | 1 | WWP1 |
| Target Scan, miRwalk | 8 | ARL2, SLA2, HTR4, TMEM135, RRAGA, SIDT2, DCTN5, FKBP1A |
| Target Scan, miRmap | 63 | MON1B, PROSC, USP6NL, ATXN7L3B, YWHAQ, ATP1B4, RAB35, UBN2, ECEL1, NBR1, CEP85, SGK1, WIPI2, ZNF362, HAS2, FKBP5, BSDC1, RASGEF1B, PID1, PTH, CCDC85C, NFS1, CA8, KBTBD2, FNTA. PDIA6, GORASP2, TTC14, NUAK2, NUDT4, UBE2V1, TXN2, VAMP8, SERINC3, ARL5B, BTRC, CHEK1, TMEM161B, DLL1, COPS2, HOXA10, DOLPP1, TMUB2, TBCID2, ZMAT3, CCDC19, KLF7, FGF7, SLC9A6, EFNB2, ARL3, XKR, CRK, PEX19, ZNF592, DCTD, KRT85, HSPG2, RNF183, LRIG2, ATP5G1, USP3 |
| Target Scan, micro-T-CDS | 198 | SALL1, FRY, CDC14A, ANLN, GHR, MKNK1, RAD23B, CCNE1, CDK8, DESI1, GCC2, SORT1, TMEM255A, RFX3, CPEB3, CDK17, PLD1, MOB4, FRYL, PNPLA6, UBE2W, ASH1L, SH2D2A, ACVR2A, RBBP6, PPM1H, KIAA0226L, YWHAH, RAB9B, EPHA7, WBP11, BCL7A, TGIF2, CCND1, RUNX1T1, DMTF1, PTPN4, HIPK3, VEGFA, ELL, KIF1B, SMURF2, TNRC6B, PNISR, CDC27, ALOX12, HECTD1, ISLR, IPPK, IKBKB, SESN1, MYLK, DZIP1, ACSBG1, DDX3Y, AKAP12, MEOX2, PHACTR2, RSBN1, PI4K2B, TMEM74B, TMEM189-UBE2V1, SHOC2, FBXW7, SSR1, ESRP1, TRPM3, ISOC1, CDC23, ZMYM2, RNF10, N4BP1, C20orf194, RASSF8, HOXC11, RAP2C, GPR63, MED26, UBFD1, SKIL, POLR3F, SOBP, ANO3, AP5S1, ZSWIM3, PLEKHA5, AMOT, MT2A, LPHN2, ZBTB46, HNRNPA1, AKT3, PARD6B, COL12A1, BTAF1, PCDHA6, MOB3B, CBFA2T3, MYB, ANKS1A, COBLL1, PVRL1, WASL, GNAI3, EXOC5, STRADB, BMPR1A, PRKAR2A, SYT10, PAFAH1B1, PCMT1, TFCP2L1, SLC2A3, PAG1, SOS2, ADORA2A, LIPE, SIRT4, FAM135A, SIPA1L2, PRDM4, PLXNC1, SLC12A2, C1orf21, PTGFR, PLAGL1, PIM1, GOLGA1, AGO4, CDC37L1, EYA1, VAPB, PRKAB2, RAF1, WWC1, SYNRG, ARPP19, SMAD7, EXOC3L2, BZW1, PHF19, TFAP2D, PLEKHA1, STK33, GALNT7, CCNT2, RNF138, CCND3, KIF3B, CHAC1, DYRK1B, LHX1, SLC7A2, FERMT2, CLOCK, AGO1, RTN4, FBXO21, SYNDIG1, BTN1A1, LRP6, EZH1, RORA, SLC22A17, DNAJA2, CPEB2, SOX5, CAPRIN1, RREB1, DCUN1D1, DCUN1D4, TBPL1, TNFSF13B, RNF144B, BFAR, RECK, UBE4B, SLC41A2, SALL4, CYP26B1, RAB30, FAM189A1, ACVR2B, HIPK2, CCND2, PTPN3, YAP1, C2orf42, TLL1, ELL2, SPAG7, VTI1B, HMGA1, NXPH1, KCNN4, E2F3, USP44, MIB1 |
| miRmap miRwalk | 28 | POLR2G, TSC22D1, GSS, CIAO1, DUSP18, MTG1, FBXL20, CHMP3, GPAM, TMEM229B, PLEKHJ1, RNF114, C11H2orf42, HSPBAP1, AHCY, LEPROT, KLHDC8B, TMEM9B, THRSP, RAB11B, TAT, OS9, SPSB3, RPUSD4, TMEM141, BRF2, HEYL, CDK10 |
| miRwalk micro-T-CDS | 14 | EED, TRAM1, CLCN4, TASP1, TCP1, LMAN2L, TTLL9, GDPD2, AGO3, URI1, CS, RBMS2, CDH1, WDR18 |
| miRmap micro-T-CDS | 22 | LLGL2, STARD13, SCRN1, LAMTOR3, ANKRD17, MRPS9, NFATC3, PIGT, SMARCA1, CD99L2, RAPGEF2, NUDCD3, ACP2, PTK7, FLNB, MADD, SLC4A7, BCL2L13, KDR, STX1A, MTMR3, SMAD5 |

Supplementary Table S7: List of miRNA target predicted by various tools for miRNA (bta-miR-1246)

| Name of Target prediction tool | Targets count | Genes |
| --- | --- | --- |
| Target Scan miRmap miRwalk | 14 | TMEM39A, POGLUT1, PNMA2, HINFP, LIN7C, ANKRA2, TAGLN2, MYNN, NHEJ1, INA, CREG1, COL2A1, GAD1, NCOA4 |
| Target Scan miRwalk micro-T-CDS | 5 | BRCC3, NTRK2, SPA17, ARL5A, MEF2C |
| Target Scan miRmap micro-T-CDS | 26 | LRFN5, ZNF583, CYSTM1, MEIS1, SEPHS1, EIF4E, GYS2, SLC17A6, ZFP36L1, SEMA6D, PPP1R11, NUDT4, EIF4EBP2, MBNL2, SLC16A7, CADM2, BCL2L2, GOLGA1, SLC38A2, CCNT2, EIF2AK3, SEMA6A, CREBL2, KRR1, DYNC1I1, PTPLAD2 |
| miRmap miRwalk micro-T-CDS | 5 | RNF4, MEGF10, CHMP3, IRAK2, CMTM6 |
| Target Scan miRwalk | 22 | ADAM12, QKI, CHMP2B, DSTYK, GNS, FAM107B, SLC25A15, POSTN, SLC37A2, DIABLO, STAM2, HGF, HYAL1, MYADM, DCK, HSPBAP1, RCAN2, F11, SLC26A11, OPALIN, NT5C3A, ACSL6 |
| Target Scan miRmap | 119 | DMP1, CCNI, SH3BP4, DKK2, C1QTNF3, RTF1, EVI2A, GSG2, SYT13, PPM1A, GNAT1, ERLIN2, EDNRA, UNC13B, DPYD, CLDN6, GORAB, ATP6V1C1, PGM2L1, ZNF667, ARHGAP42, HARS, RANBP1, PARD6G, TCF19, GRPEL2, FSHR, ACLY, OSTN, WDR38, PIGT, CKS2, GRAMD1C, B3GALT2, TSPAN12, NRN1, RCHY1, SESN1, CLPTM1L, FKBP3, ATXN7L3, CYTIP, DNAH10, NAB1, MAP3K7, FBXL3, OLFML3, AGPAT5, RAB9A, SUN3, ZMPSTE24, TLK2, SH3GL2, SUCLG2, HNRNPA1, SLC16A1, FAM45A, SF3B3, ARL8B, PIGM, WLS, DHCR24, C1R, CALB1, GPR116, NFATC1, WDR1, REEP3, DCAF12, TCHHL1, KRAS, VPS36, ITGA8, ZNF566, LEPROT, CHST4, BEST1, ZRANB1, CHUK, PCK1, OXR1, HEXB, PTER, NEUROD1, RDH16, TRIM9, LIX1, AADAT, HEG1, PRR11, GAPT, LAMP5, FERMT2, P2RY13, TPRG1, IL2RA, PPEF1, TAT, ACSM2A, BRI3, STX12, IQGAP1, SCRN3, PTS, METTL7A, MBLAC2, TLR4, CRK, NPY2R, ORC6, YIPF5, RFX4, LRRC16A, IL20RB, ZZZ3, CASQ2, DNAJC24, THAP6, GOLT1B |
| Target Scan micro-T-CDS | 143 | MITF, TAF9B, TEAD1, UTRN, ZNF83, FAM98A, SIAH3, HECTD2, MMP16, FAXC, PIK3AP1, GNRHR, KIAA0355, KDM5A, TMEM33, ZNF227, HOXD13, GMFB, SLAIN1, AP3B1, DCX, FSTL5, NHLH2, GRIA1, C11orf44, FBXO3, PLCXD3, LUZP2, KCNAB1, FAM122B, CLLU1, C3orf36, NRXN1, CGRRF1, ISCA1, GSK3B, ANGPTL1, GSG1L, BRI3BP, ZNF528, MPZ, VKORC1L1, MMP8, RAD9B, DDI1, KLHL15, PAPOLG, RASA2, C4orf32, LRRTM2, MUT, AGL, ARFIP1, ACVR1, MC2R, FER, DMRT2, KIAA0895, CXorf27, METAP2, C17orf102, ZSCAN31, GAS1, PURB, GTF2I, LAMP2, RGS17, PCBP2, PIP5K1B, SKIL, PYGO1, NCOA7, GPR139, CCER1, FBXO28, CTXN3, RGS7BP, ZFP42, FAM13B, CCDC178, ANKRD34C, ZC3H12B, SOX2, PSD3, MCTP1, ESM1, RBBP9, FEM1B, DHX33, GPATCH2L, FCRL3, ATP6V1G3, CDH2, EXOSC10, ARL4C, PLEKHG2, GPALPP1, EXOSC9, GPR12, WT1, TMEM132C, TMTC3, VIM, KIAA1244, FAM53C, BHLHB9, WASF3, PABPC4L, EFHC2, FGF14, PTAR1, GPR26, B3GAT2, DGKI, ZNF23, CT62, ZKSCAN8, SRPK2, FUT9, PMP2, ANKFY1, EDA, LDB2, NECAB1, QTRTD1, TNFRSF11A, PRLR, CFTR, PA2G4, PDE11A, CLVS1, ZNF470, GLRB, DYRK1A, ZNF800, KCTD16, LHFPL3, ZNF770, GMNC, ONECUT2, NDFIP1, WAC, ST8SIA6 |
| miRmap miRwalk | 33 | FIBIN, BET1L, TERF2IP, SETMAR, CNGA2, JAGN1, OTUB2, BPNT1, BNIP1, OAS2, TMEM229B, SNX15, PIPOX, RAB3C, PKD2, NIPSNAP1, MTF1, FAM110A, SNAP25, KLHDC8B, P4HTM, KCNMB1, LASP1, UBL3, SNAP23, S1PR1, AP3M1, CEBPG, ZMYND11, ATP6V0D2, CYP2B6, ALG10, CCDC115 |
| miRwalk micro-T-CDS | 18 | DUSP18, LMAN2L, SOCS4, SNAPC5, DIO2, TSPYL1, GPR85, CFLAR, FAM71D, DTNA, POLA2, CWC27, ACO1, SMAD4, CPT2, FXR1, NFIB, UBE2D2 |
| miRmap micro-T-CDS | 47 | SORL1, USP6NL, SDAD1, HS3ST1, FIGF, UBN2, ACVR2A, PAQR3, SLC39A9, AKAP2, GTF2H5, ARHGAP26, PRKACB, ALDH5A1, VPS35, TMEM182, WDR77, RC3H2, UBE2Z, RAPGEF2, SMG7, SFT2D2, BCL2L14, TMEM130, FAM151B, UNC5B, YTHDF3, ZNF148, POU2AF1, GMCL1, SLC26A3, ITSN2, CCNG2, DLG3, Mar-03, PPHLN1, PLEKHA3, GDAP1, TNFSF18, CSRNP3, PTPRD, CAPZA1, TBCA, JARID2, AQP1, TRPS1, SGPP1 |

Supplementary Table S8: List of miRNA target predicted by various tools for miRNA (bta-miR-199B)

| Name of Target prediction tool | Targets count | Genes |
| --- | --- | --- |
| Target Scan miRmap miRwalk | 2 | LASP1, ATP1B3 |
| Target Scan miRwalk micro-T-CDS | 1 | AGO3 |
| Target Scan miRmap micro-T-CDS | 14 | SORL1, ZNF148, JAG1, MCFD2, WIPI2, KLHL29, MATN2, FZD6, ZFYVE27, SIRT1, CLIP1, LIN7C, RANBP2, MAP3K11 |
| Target Scan miRwalk | 4 | HSPBAP1, ABCA1, FXR1, HGF |
| Target Scan miRmap | 38 | NUFIP2, TMEM19, APPBP2, ACTR1A, UNG, FBXO33, MAGT1, CDCA7L, KIT, ATG4D, FKBP5, OSTM1, DERL2, M6PR, ARHGEF5, RANBP3, LMAN2, USP46, DDX6, SRSF1, ARHGAP29, EHD4, MAPK8, ERLIN1, CDKN1C, SERPINE1, TSPAN6, NBL1, SLC35A3, ZMAT2, SMARCAD1, ARHGAP35, RNF8, PMP22, LEPREL1, TMEM215, CHCHD4, DPP10 |
| Target Scan micro-T-CDS | 184 | ZNF225, RNF38, PVRL1, FAM178A, RGMB, TGFB2, UBE2G1, RASSF3, SLC25A37, AP1G1, NINL, CYLC2, ARHGAP21, POU3F1, VPS26A, ZNF704, TENM4, SRRM4, ANO5, KIAA1958, RAD23B, KLHL23, MGAT4B, KIAA2018, LCOR, CDKN1B, IPO8, ACOX1, MYH9, TOX3, SOS2, MYEF2, TRAF3, HMCN1, KIAA0355, CDH2, ZBTB18, ZNF641, UBN2, PLXNC1, NAALADL2, STON2, RBPMS, FAM188A, IL23R, USPL1, ANKRD52, ECE1, YIPF6, PPARGC1A, ZNF709, SNAI1, RORB, PXN, GANAB, KCND3, ZNF516, DUSP14, DDI2, RAB9B, RBBP4, EXOC8, EPHA7, CACUL1, ZNF627, AQP11, PCYOX1, PPFIBP1, ZNF614, ATXN7, C1GALT1, GCNT2, ARHGAP12, PODXL, PDE4D, ASRGL1, B3GNT1, TMEM63B, KLF9, FIGN, ABHD17C, NAA40, TSPAN5, FP15737, ZNF846, SRRM1, GPR89B, KIAA1109, ITGA4, PRPF40A, CLOCK, AGO1, NPAS2, PDPN, PHACTR4, ZBTB42, ZBTB37, TMEM178B, BTBD3, PPP1R2, GIT1, KPNA4, CSDC2, EIF5B, SACS, MN1, CCNL1, MYRF, AKAP1, CCNJ, UBL3, CAPRIN1, ZNF23, SP1, SEC24C, TMED8, VGLL2, ZNF226, SORCS3, ZKSCAN5, BRD7, CEP85L, ZNF544, SLC35E1, RASSF2, RBM47, STK4, ZNF256, ETS1, CDKN2AIP, FER, WDR44, BICC1, WNK3, RB1, CDC73, ZFP2, TST, LYSMD3, PAN3, SHOC2, CLCN3, ACVR2B, FLRT3, ZDHHC21, PPP6C, SRGAP3, SULF1, SUCO, HHIP, ZNF512B, MGAT3, RGS10, MRPS25, RALGAPA1, ZNF654, ABCC1, NSG1, ZNF791, AFTPH, LARP4, SLC24A3, RLIM, GPR89A, DDR1, ZNF579, RNF11, BEND3, KPNB1, FZD4, MFHAS1, ONECUT2, AUTS2, SLC25A23, DDX3X, TAOK1, MAB21L1, HSPA12A, EVX2, SAT1, HDLBP, ZNF788, HAPLN1, ZNF652 |
| miRmap miRwalk | 7 | NLRP5, LEPROT, EEPD1, UTP14A, SRSF2, PLEKHJ1, PDHB |
| miRwalk micro-T-CDS | 12 | MITF, DCK, ILF2, LSM14A, SLX1A, FETUB, THY1, AADAT, CCR7, MED4, HSPA9, RAD21 |
| miRmap micro-T-CDS | 12 | RGS16, GPN2, ACP2, ZNF527, MEGF10, IRAK2, SEC22C, ARHGEF15, GLYR1, TMEM97, TMEM168, GJA5 |

Supplementary Table S9: List of miRNA target predicted by various tools for miRNA (bta-miR-339B)

| Name of Target prediction tool | Targets count | Genes |
| --- | --- | --- |
| miRmap miRwalk micro-T-CDS | 3 | RAB15, TMEM119, RFC5 |
| miRmap miRwalk | 59 | MTA2, RDH13, C11H2orf49, MGC152281, BIRC5, C17H12orf43, MAPKAP1, SGPL1, MYADML2, KIAA1143, STIM1, TBC1D22B, STK40, CHMP7, MTTP, TRAPPC2L, TMEM25, VAPB, DNAJC11, COQ10A, PDGFB, CPT1B, CCDC86, KXD1, KCNJ10, EDEM2, RAB7B, TSPAN2, CTSF, FAF2, BSG, LAMP5, CCR4, TK1, MRAS, SLC31A1, SCAMP4, TMEM182, GULO, NECAP1, BRI3, SAP18, SLC38A3, ABAT, SYT11, MCC, RHOJ, RASL10A, POLR3H, RANBP10, ALDP, SPSB1, SPRY4, GIMAP5, MASP1, PLA2G12A, MLPH, ABHD6, BAIAP2, HCLS1 |
| miRwalk micro-T-CDS | 26 | SLC35F6, ERLIN2, EMX2, TMEM79, KLHL21, TMEM169, CWH43, IQCB1, TSPAN14, ECI2, CCR7, RANBP3, FLCN, GDAP2, ISLR, PEA15, VPS4B, RASL12, TFAP4, ZDHHC9, TNPO3, SFTPB, ERI3, RBM27, UBE3A, HNRNPA1 |
| miRmap micro-T-CDS | 37 | FARP1, IRAK1, SP140L, SHC1, ALKBH1, EVC2, PAK6, ACTR1A, PRKAA1, AKIRIN1, CNNM4, HNRNPK, NCK2, ABCG4, PODXL, ZNRF2, VCP, M6PR, FBXO30, ACSS2, RFC2, ZC3HC1, SORBS1, KREMEN1, PPIL2, ERLIN1, KLHL29, BRMS1L, TMOD1, PLEKHH3, ARHGAP36, ANK3, F11R, MLLT11, USP8, GPR61, MOB3B |

Supplementary Table S10: Gene Ontology and pathway analysis of selected miRNAs (bta-miR-1246)

| Term | Count | P Value | | | Genes | | |
| --- | --- | --- | --- | --- | --- | --- | --- |
| Biological processes of bta-miR-1246 | | | | | | | |
| GO:0071320~cellular response to cAMP | 7 | | 6.25E-05 | | | WT1, RAPGEF2, PKD2, SLC26A3, CFTR, AQP1, ZFP36L1 | |
| GO:0015031~protein transport | 15 | | 5.44E-03 | | | JAGN1, LMAN2L, GOLT1B, YIPF5, CMTM6, LIN7C, SDAD1, BET1L, CHMP2B, CHMP3, SFT2D2, STAM2, ARL3B, CFTR, AXDND1 | |
| GO:0051092~positive regulation of NF-kappaB transcription factor activity | 9 | | 0.003884 | | | FER, EDA, CHUK, DHX33, IRAK2, TERF2IP, TNFRSF11A, TLR4, BCL2L2 | |
| GO:0008283~cell proliferation | 7 | | 0.022279 | | | PURB, SMAD4, FER, CKS2, S1PR1, GNAT1, ZFP36L1 | |
| GO:0007034~vacuolar transport | 3 | | 0.011734 | | | NDFIP1, CHMP3, ATP6V0D2 | |
|  |  | |  | | |  | |
| GO:0007252~I-kappaB phosphorylation | 3 | | 0.014874 | | | CHUK, MAP3K7, TLR4 | |
| Cellular processes of bta-miR-1246 | | | | | | | |
| GO:0005634~nucleus | 110 | | 6.92E-06 | | | CCNT2, CCNI, UBE2D2, NAB1, DMP1, UBE2Z, AQP1, NUDT4, FAM110A, SOX2, ZSCAN31, SESN1, ZC3H12B, PAPOLG, PABPC4L, FAM53C, PRKACB, VPS36, GTF2I, MEF2C, MBNL2, TSPYL1, DYRK1A, SETMAR, MITF, RC3H2, WDR77, PYGO1, RFX4, GMNC, PPEF1, ANKRA2, PRR11, FKBP3, DGKI, CFTR, KLHL15, CREBL2, IQGAP1, BRCC3, ZNF23, GMCL1, ZFP36L1, NEUROD1, FXR1, IRAK2, ZKSCAN8, PCBP2, ZNF148, SKIL, NHEJ1, SRPK2, ZRANB1, SMAD4, DHCR24, NFATC1, QKI, FER, POLA2, FGF14, RCAN2, FBXL3, KDM5A, GSK3B, ZNF770, CSRNP3, PPP1R11, ONECUT2, DUSP18, LDB2, CALB1, TLK2, JARID2, TEAD1, TCF19, CHUK, DCK, PPM1A, MTF1, WAC, TERF2IP, NCOA7, PMP2, RGS7BP, OTUB2, SF3B3, ZNF470, CEBPG, PARD6G, RCHY1, PURB, UBN2, CREG1, SH3BP4, ZNF227, HNRNPA1, TAF9B, EIF4E, DYNC1I1, ZNF583, FEM1B, DMRT2, AGL, OXR1, HINFP, MEIS1, NFIB, CCNG2, VIM | |
| GO:0005737~cytoplasm | 99 | | 3.84E-04 | | | CCNI, DMP1, NUDT4, FAM110A, PTAR1, CDH2, SESN1, SPA17, SEPHS1, PRKACB, MAP3K7, SH3GL2, GTF2I, MEF2C, MBNL2, DYRK1A, MITF, WDR77, LIX1, ZFP42, UTRN, PRR11, DNAJC24, IQGAP1, BRCC3, LIN7C, NEUROD1, FXR1, MC2R, CYP2B6, IRAK2, PCBP2, EIF4EBP2, SLC38A2, BCL2L14, SRPK2, ZRANB1, SMAD4, DNAH10, DTNA, GPR12, SIAH3, TPRG1, KLHDC8B, DHCR24, NFATC1, ARHGAP26, HSPBAP1, QKI, FER, FGF14, DLG3, RCAN2, ACO1, BCL2L2, GSK3B, SNAP25, WDR1, DUSP18, GYS2, TRIM9, DSTYK, HYAL1, CHUK, DCK, NT5C3A, COL2A1, OAS2, MTF1, DPYD, DCX, RAPGEF2, TERF2IP, PMP2, RGS7BP, FAXC, PARD6G, RNF4, EXOSC9, S1PR1, SH3BP4, HNRNPA1, DCAF12, EIF4E, WASF3, DYNC1I1, RANBP1, YTHDF3, KRR1, AGL, CFLAR, GTF2H5, ACVR2A, GLRB, WT1, CCNG2, KRAS, VIM, METAP2 | |
| GO:0005654~nucleoplasm | 59 | | 0.001711 | | | GSK3B, ONECUT2, GORAB, SNAP23, DUSP18, LDB2, UBE2Z, CWC27, CPT2, TRPS1, SESN1, FBXO3, SLC16A7, SLC26A11, CYTIP, TEAD1, GTF2I, TSPYL1, MBNL2, CHUK, PPHLN1, CGRRF1, DCK, WDR77, ACLY, OAS2, MTF1, WAC, ZMYND11, UTRN, STAM2, STX12, SF3B3, MYNN, HOXD13, BRCC3, SDAD1, RCHY1, PURB, ORC6, EXOSC10, DHX33, EXOSC9, UBN2, PCBP2, S1PR1, RBBP9, HNRNPA1, ZNF148, NHEJ1, ZRANB1, SMAD4, DTNA, SIAH3, KRR1, FEM1B, YIPF5, NFIB, FERMT2 | |
| GO:0016020~membrane | 34 | | 0.001855 | | | SNAP25, GOLT1B, HEXB, PARD6G, PKD2, FXR1, CYP2B6, CNGA2, SLC26A11, SPA17, SH3GL2, ACSL6, DIO2, LHFPL3, DHCR24, PRLR, SGPP1, RC3H2, PPM1A, OAS2, IL2RA, REEP3, FSHR, RAPGEF2, TMEM39A, KRAS, CYSTM1, CLPTM1L, ANKRA2, CRK, DGKI, CFTR, BCL2L2, TMEM229B | |
| GO:0098978~glutamatergic synapse | 14 | | 2.88E-04 | | | GRIA1, GSK3B, SNAP25, PTPRD, FXR1, LRFN5, CALB1, DLG3, GSG1L, RGS7BP, EIF4E, SH3GL2, DGKI, WASF3 | |
| Molecular functions of bta-miR-1246 | | | | | | | |
| GO:0046872~metal ion binding | 55 | | | 5.48E-06 | | | KDM5A, ISCA1, LASP1, ZC3H12B, PAPOLG, ANKFY1, GNAT1, ZNF566, SEPHS1, ACVR1, TCF19, POSTN, MBNL2, CGRRF1, QTRTD1, PRLR, PGM2L1, RC3H2, PYGO1, ACLY, MBLAC2, COL2A1, OAS2, DPYD, RASA2, ZMYND11, LMAN2L, ZNF470, NRXN1, MYNN, DNAJC24, ZNF23, BRCC3, GNS, PTS, ZMPSTE24, ZFP36L1, PDE11A, ZKSCAN8, BPNT1, ZNF667, ZNF227, ZNF148, PCK1, ZNF583, ZRANB1, SMAD4, DMRT2, SIAH3, B3GAT2, ACVR2A, HINFP, WT1, ACO1, METAP2 |
| GO:0000978~RNA polymerase II core promoter proximal region sequence-specific DNA binding | 29 | | | 0.019517 | | | ZNF770, ZNF470, ONECUT2, MYNN, HOXD13, ZNF23, NEUROD1, NHLH2, ZSCAN31, ZKSCAN8, ZNF227, ZNF667, ZNF148, SKIL, TEAD1, ZNF583, SMAD4, MEF2C, DMRT2, MITF, NFATC1, HINFP, MEIS1, NFIB, WT1, RFX4, MTF1, ZFP42 |
| GO:0003677~DNA binding | 27 | | | 7.10E-03 | | | KDM5A, ZNF470, ONECUT2, CEBPG, CREBL2, NEUROD1, SOX2, ORC6, ZNF566, ZNF148, JARID2, TEAD1, GTF2I, ZNF583, SMAD4, MEF2C, MITF, NFATC1, DCK, RC3H2, HINFP, THAP6, POLA2, MEIS1, NFIB, POU2AF1, BCL2L2 |
| GO:0001228~transcriptional activator activity, RNA polymerase II transcription regulatory region sequence-specific binding | 21 | | | 5.91E-05 | | | ZNF770, SMAD4, MEF2C, CSRNP3, ZNF470, ONECUT2, DMRT2, MITF, HOXD13, HINFP, NEUROD1, NHLH2, MEIS1, NFIB, WT1, RFX4, MTF1, ZKSCAN8, ZNF227, GTF2I |
| GO:0003682~chromatin binding | 13 | | | 0.047104 | | | SMAD4, TSPYL1, CCNT2, MITF, HOXD13, HINFP, NEUROD1, MEIS1, CKS2, WAC, GMNC, JARID2, SKIL |
| KEGG pathway analysis for bta-miR-1246 | | | | | | | |
| Bta04151:PI3K-Akt signaling pathway | 14 | | | 0.058139 | | | NTRK2, GSK3B, CHUK, HGF, PRLR, GYS2, FGF7, IL2RA, ITGA8, KRAS, PCK1, PIK3AP1, TLR4, EIF4E |
| bta04010: MAPK signaling pathway | 12 | | | 0.045378 | | | NTRK2, PPM1A, MEF2C, CHUK, HGF, RASA2, GIFR, KRAS, NFATC1, MAP3K7, PRKACB, CRK |
| bta04144: Endocytosis | 11 | | | 0.039152 | | | IL2RA, CAPZA1, PSD3, CHMP2B, PARD6G, CHMP3, PIP5K1B, VPS35, VPS36, STAM2, SH3GL2 |
| Signaling pathways regulating pluripotency of stem cells | 9 | | | 1.12E-02 | | | ACVR1, SOX2, GSK3B, SMAD4, MEIS1, KRAS, JARID2, SKIL, ACVR2A |
| Measles | 9 | | | 2.01E-02 | | | GSK3B, CHUK, OAS2, IL2RA, EIF2AK3, RAB9A, RCHY1, MAP3K7, TLR4 |

Supplementary Table S11: Gene Ontology and pathway analysis of selected miRNAs (bta-miR-195)

| Term | | Count | P Value | Genes |
| --- | --- | --- | --- | --- |
| Cellular component of bta-miR-195 | | | | |
| GO:0005737~cytoplasm | | 114 | 9.42E-11 | PID1, PRDM4, WWC1, BZW1, CDC14A, NUDT4, MYLK, LIPE, PCMT1, MT2A, CCND3, FGF7, CDC23, SALL1, CCND2, FNTA, CCND1, CDH1, SESN1, CHEK1, CDC27, PIM1, CHAC1, BTRC, ANKS1A, SH3GL2, PRKAB2, USP3, UBE4B, HSPG2, MOB3B, NRBP1, CCNE1, MADD, TBPL1, RAF1, FKBP5, LLGL2, ANKRD17, PTGFR, URI1, MTMR3, ARL3, ARL2, BART, ATXN7L3B, PLD1, ARPP19, CIAO1, STK33, IRAK2, PRKAR2A, ISOC1, TASP1, CDK17, STARD13, SMURF2, BSDC1, KLHDC8B, HSPBAP1, SMAD5, SMAD7, MOB4, CAPRIN1, COPS2, CDK10, CPEB3, DZIP1, CPEB2, BCL2L2, RNF10, MTMR11, TFCP2L1, DUSP18, SIPA1L2, MEOX2, IKBKB, NUDCD3, YWHAQ, SCRN1, CA8, YWHAH, TSC22D1, USP44, RNF144B, RAPGEF2, ACSBG1, PAFAH1B1, YAP1, KANK1, PPM1H, FBXL20, MAPK8, PARD6B, MKNK1, CDC37L1, SHOC2, HNRNPA1, ARL2, ARL3, PEX19, PLEKHA1, EYA1, XPO7, WWP1, HIPK3, ACVR2A, HIPK2, VEGFA, MIB1, FKBP1A, HEYL, THRSP, PTPN4, TTLL9, PTPN3 |
| GO:0005634~nucleus | | 107 | 1.19E-07 | CCNT2, PRDM4, WWC1, RORA, DCAF7, NUDT4, HOXA10, LIPE, MT2A, CCND3, SALL1, RNF114, CCND2, CCND1, SESN1, SALL4, MYB, CDC27, KDR, BTRC, SOX5, PRKAB2, USP3, DYRK1B, RFX3, C11H2ORF42, UBE4B, RSBN1, MOB3B, RRAGA, DMTF1, CCNE1, UBE2V1, RAF1, IPPK, ANKRD17, URI1, ARL3, DCUN1D4, ARL2, CREBL2, STK33, KIF3B, IRAK2, ZNF423, RREB1, SKIL, N4BP1, CDK17, TFAP2D, HMGA1, NFATC3, SMARCA1, SMAD5, SMAD7, CDK8, UBE2W, AGO4, LHX1, CDK10, CPEB3, CPEB2, PHF19, RNF10, TFCP2L1, DUSP18, WASL, SOBP, MEOX2, HOXC11, IKBKB, JARID2, RBM6, TBP, TSC22D1, USP44, STRADB, PPP2R5C, ASH1L, CBFA2T3, RPUSD4, SGK1, RBMS2, YAP1, ZBTB46, NFS1, MAPK8, PARD6B, BTAF1, MKNK1, UBN2, HAS2, E2F3, RBBP6, SHOC2, POLR2G, HNRNPA1, RUNX1T1, SPAG7, BRF2, PEX19, EYA1, XPO7, HIPK3, HIPK2, HEYL, THRSP |
| GO:0005654~nucleoplasm | | 65 | 1.66E-06 | GPR63, GCC2, DUSP18, SLA2, HOXC11, AMOT, CCND3, CCND2, CCND1, SESN1, SALL4, CHEK1, MYB, CDC27, PIM1, ANKS1A, AP5S1, TGIF2, FBXW7, USP3, DYRK1B, C11H2ORF42, PPP2R5C, ASH1L, WIPI2, RAD23B, CBFA2T3, RPUSD4, DMTF1, CCNE1, ESRP1, NUP50, UBE2V1, CLOCK, FKBP5, ANKRD17, DCTN5, URI1, ARL3, DCUN1D1, PPM1H, ARL2, NFS1, BTAF1, ZMAT3, UBN2, PLAGL1, SHOC2, HNRNPA1, RAB11FIP2, RUNX1T1, PEX19, PLEKHA1, PLEKHA5, NFATC3, SMARCA1, SMAD5, SMAD7, ANLN, KLF7, AGO3, THRSP, CPEB3, FERMT2, TNRC6B |
| GO:0005829~cytosol | | 78 | 1.13E-05 | CCNT2, ELL, WWC1, GPR63, GCC2, WASL, HOXC11, CDC14A, GHR, AKAP12, IKBKB, LIPE, PPP6C, PCMT1, RNF114, CCND2, MYB, PIM1, CHAC1, ANKS1A, AP5S1, PLEKHJ1, PNISR, DESI1, EED, STRADB, PPP2R5C, WIPI2, RAD23B, VAMP8, RAP2C, DMTF1, MADD, CHMP3, UBE2V1, SGK1, RAF1, CLOCK, RBMS2, LLGL2, PAFAH1B1, YAP1, URI1, AHCY, DCUN1D1, ARL2, ALOX12, NFS1, PARD6B, FLNB, SHOC2, N4BP1, VTI1B, PEX19, PLEKHA1, SORT1, GSS, PLEKHA5, NFATC3, KLHDC8B, SMAD5, HIPK3, SMAD7, FKBP1A, KLF7, AGO3, AGO4, CAPRIN1, AGO1, DNAJA2, NBR1, THRSP, CEP85, CPEB3, DZIP1, FERMT2, BCL2L2, RAB9B |
| GO:0005794~Golgi apparatus | | 26 | 5.51E-04 | ARL3, ARL2, GCC2, SLA2, PLD1, NAPG, LRP6, FGF7, CDH1, PLAGL1, GOLGA1, KDR, HAS2, VTI1B, GALNT7, RNF24, SORT1, SEMA6D, PPP2R5C, ASH1L, CLCN4, GORASP2, VAPB, TCP1, CEP85, RAF1 |
| Biological processes of bta-miR-195 | | | | |
| GO:0006468~protein phosphorylation | 14 | | 1.89E-04 | CDK17, ACVR2B, SMAD5, ACVR2A, MYLK, IKBKB, CDK8, STK33, IRAK2, CCNE1, PIM1, CDK10, RAF1, BMPR1A |
| GO:0051301~cell division | 11 | | 0.002906 | CCND3, PARD6B, SEH1L, CDC23, CCND2, CCND1, CDC27, GNAI3, WASL, ARPP19, PAFAH1B1 |
| GO:0045893~positive regulation of transcription, DNA-templated | 15 | | 0.002419 | HMGA1, DYRK1B, RFX3, CREBL2, RAP2C, FGF7, CDH1, LHX1, PIM1, TASP1, NDP, BTRC, ZNF423, ATXN7L3, YWHAH |
| GO:0048227~plasma membrane to endosome transport | 3 | | 0.005085 | USP6NL, SORT1, RAB35 |
| Molecular functions of bta-miR-195 | | | | |
| GO:0004674~protein serine/threonine kinase activity | 17 | | 5.24E-05 | DYRK1B, ACVR2B, HIPK3, ACVR2A, HIPK2, IKBKB, MAPK8, STK33, IRAK2, MKNK1, AKT3, CHEK1, PIM1, CDK10, SGK1, RAF1, BMPR1A |
| GO:0004672~protein kinase activity | 13 | | 4.13E-05 | CDK17, STRADB, HIPK3, HIPK2, MYLK, IKBKB, FXL, NUAK2, CCND1, PTK7, IRAK2, PIM1, RAF1 |
| GO:0046332~SMAD binding | 6 | | 4.91E-04 | FKBP1A, SMURF2, SKIL, FERMT2, BMPR1A, NDP |
| GO:0019003~GDP binding | 7 | | 6.75E-04 | RAP2C, ARL3, RAB35, GNAI3, ARL2, RAB9B, RAB11B |
| KEGG pathway analysis for bta-miR-195 | | | | |
| bta04390: Hippo signaling pathway | 14 | | 9.66E-06 | YAP1, WWC1, SMAD7, AMOT, CCND3, PARD6B, CCND2, CCND1, YWHAQ, CDH1, BTRC, BMPR1A, YWHAH, LLGL2 |
| bta04151:PI3K-Akt signaling pathway | 17 | | 0.002742 | PPP2R5C, VEGFA, AKT, IKBKB, CCND3, FGF7, CCND2, CCND1, YWHAQ, CCNE1, AKT3, MYB, KDR, SGK1, LCRMP1, SOS2, YWHAH |
| bta05166: Human T-cell leukemia virus 1 infection | 14 | | 7.08E-04 | TBP, NFATC3, IKBKB, CCND3, MAPK8, CDC23, CCND2, CCND1, CCNE1, AKT3, CHEK1, CDC27, TBPL1, E2F3 |
| bta04218:Cellular senescence | 12 | | 3.49E-04 | CCND3, CCND2, CCND1, CCNE1, AKT3, CHEK1, NFATC3, E2F3, BTRC, RAF1, HIPK3, HIPK2 |

Supplementary Table S12: Gene Ontology and pathway analysis of selected miRNAs (bta-miR-339b)

| Term | Count | P Value | Genes |
| --- | --- | --- | --- |
| Biological processes of bta-miR-339b | | | |
| GO:0030433~ubiquitin-dependent ERAD pathway | 4 | 0.012549 | VCP, FAF2, ERLIN1, TSPAN |
| GO:0006397~mRNA processing | 4 | 0.03655 | P53, HNRNPK, SAP18, HNRNPA1 |
| GO:0045541~negative regulation of cholesterol biosynthetic process | 2 | 0.02552 | ERLIN1, ERLIN2 |
| GO:0032510~endosome to lysosome transport via multivesicular body sorting pathway | 2 | 0.031798 | VCP, VPS4B |
| GO:0032933~SREBP signaling pathway | 2 | 0.056513 | ERLIN1, ERLIN2 |
| Cellular processes of bta-miR-339b | | | |
| GO:0005654~nucleoplasm | 27 | 1.09E-04 | SLC35F6, VCP, ACSS2, MAPKAP1, ZNRF2, IRAK1, ALKBH1, PEA15, PAK6, TSPAN2, HNRNPA1, MTA2, IQCB1, AKIRIN1, RANBP3, PPIL2, MCC, ALDP, alda, ZC3HC1, MLLT11, HNRNPK, TFAP4, CCDC86, POLR3H, MASP1, CHMP7, STK40 |
| GO:0005829~cytosol | 26 | 0.017363 | SLC35F6, VCP, PRKAA1, ACSS2, VPS4B, MAPKAP1, UBE3A, FLCN, IRAK1, PEA15, NCK2, USP8, TRAPPC2L, SPRY4, ERLIN2, MCC, BAIAP2, MLLT11, SPSB1, ZDHHC9, RANBP10, BIRC5, HCLS1, MASP1, CHMP7, STK40 |
| GO:0005783~endoplasmic reticulum | 11 | 0.00471 | SGPL1, VCP, TRAPPC2L, ZDHHC9, STIM1, ALKBH1, MTTP, NCK2, TMEM119, ERLIN1, CWH43 |
| GO:0005765~lysosomal membrane | 9 | 7.39E-06 | LAMP5, SLC35F6, FLCN, BRI3, TMEM79, M6PR, KXD1, ABHD6, CHMP7 |
| GO:0032991~macromolecular complex | 7 | 0.009031 | ZNRF2, IRAK1, ERLIN1, ABAT, ERLIN2, F11R, CNNM4 |
| Molecular functions of bta-miR-339b | | | |
| GO:0042803~protein homodimerization activity | 9 | 0.008349 | TFAP4, IRAK1, CCNB2, VPS4B, PDGFB, BIRC5, MASP1, F11R, ABCG4 |
| GO:0005525~GTP binding | 7 | 0.014086 | RASL12, MRAS, RASL10A, RAB15, RHOJ, GIMAP5, RAB7B |
| GO:0003924~GTPase activity | 6 | 0.024726 | RASL12, MRAS, RASL10A, RAB15, RHOJ, RAB7B |
| GO:0019899~enzyme binding | 5 | 0.00938 | IQCB1, FLCN, TSPAN14, VAPB, RFC2 |
| GO:0031625~ubiquitin protein ligase binding | 5 | 0.032349 | VCP, SYT11, FAF2, ERLIN1, ERLIN2 |
| KEGG pathway analysis for bta-miR-339B | | | |
| bta05132: Salmonella infection | 6 | 0.023875333 | ACTR1A, IRAK1, PODXL, RHOJ, M6PR, RABlB |
| bta04014: Ras signaling pathway | 5 | 0.049008358 | PLA2G12A, MRAS, SHC1, PDGFB, RABL2 |
| bta05211: Renal cell carcinoma | 3 | 0.043666836 | FLCN, PDGFB, PAK6 |

Supplementary Table S13: Gene Ontology and pathway analysis of selected miRNAs (bta-miR-199b)

| Term | Count | P Value | Genes |
| --- | --- | --- | --- |
| Biological processes of bta-miR-199b | | | |
| GO:0006357~regulation of transcription from RNA polymerase II promoter | 35 | 2.57E-04 | RB1, ZNF791, ONECUT2, ZBTB42, ZNF23, RORB, ETS1, NPAS2, CDKN2AIP, ATXN7, ZNF704, RAD21, ZNF527, ZKSCAN5, BRD7, ZNF148, DAZ, ZNF641, ZBTB18, MYEF2, ZFP2, ZBTB37, SMAD4, EVX2, MED4, POU3F1, TOX3, SP1, KLF9, ZNF614, SNAI1, VGLL2, ZNF654, CLOCK, ZNF652 |
| GO:0007165~signal transduction | 14 | 0.010716 | TGFB2, TENM4, SMAD2, HHIP, HGF, ARHGAP29, STK4, ARHGAP35, ARHGAP12, ARHGAP21, RASSF2, RASSF3, SHOC2, SRGAP3 |
| GO:0030335~positive regulation of cell migration | 8 | 0.006082 | FER, ONECUT2, FZD4, HGF, PDPN, KIT, SNAI1, GCNT2 |
| GO:0050680~negative regulation of epithelial cell proliferation | 6 | 3.87E-04 | RB1, CDKN1C, TGFB2, AQP11, STK4, CDC73 |
| GO:0030308~negative regulation of cell growth | 6 | 0.006653 | RB1, TGFB2, CDKN1B, DDX3X, SIRT1, CDKN2AIP |
| Cellular processes of bta-miR-199b | | | |
| GO:0005634~nucleus | 68 | 6.78E-04 | RB1, CDKN1C, RNF11, DDX3X, CCNJ, NAA40, CDKN1B, TENM4, ONECUT2, CDCA7L, HDLBP, RORB, ETS1, RASSF2, ZNF527, SACS, SMARCAD1, KPNA4, CCNL1, ZNF641, USP46, SRRM4, RALGAPA1, PDE4D, ZFP2, MITF, PRPF40A, DCK, SIRT1, TOX3, MN1, CLIP1, ZNF516, LCOR, VGLL2, KPNB1, ARHGEF5, DDX6, RBM47, ZNF791, MYRF, SRSF1, RNF8, ZBTB42, ZNF23, STK4, NPAS2, CDKN2AIP, UNG, FXR1, MAPK8, RBBP4, IRAK2, BTBD3, ZNF704, UBN2, ZKSCAN5, SHOC2, ZNF148, BRD7, PPARGC1A, ZBTB18, MYEF2, EVX2, FER, MAB21L1, RGS10, ZNF654 |
| GO:0005829~cytosol | 61 | 3.42E-06 | NAA40, CDKN1B, CDCA7L, HDLBP, STON2, SAT1, CDC73, WDR44, PPP6C, IPO8, RASSF2, RASSF3, AP1G1, GIT1, ZNF641, PLEKHJ1, USP46, PDE4D, WIPI2, RAD23B, SIRT1, TOX3, AFTPH, SRSF2, MYH9, CLOCK, KPNB1, ARHGEF5, GLYR1, DDX6, MYRF, NUFIP2, PXN, RNF8, VPS26A, STK4, CLCN3, NPAS2, FXR1, RBBP4, FLRT3, ATXN7, BTBD3, RAD21, NLRP5, SHOC2, BRD7, LARP4, FER, AGO3, OSTM1, CAPRIN1, AGO1, KLF9, WNK3, RGS10, SNAI1, RLIM, SEC24C, LSM14A, RAB9B |
| GO:0005737~cytoplasm | 60 | 0.012206 | CDKN1C, CCNJ, CDKN1B, TENM4, CDCA7L, HDLBP, STON2, ARHGAP35, IPO8, ACTR1A, RASSF2, RASSF3, CDH2, KPNA4, PHACTR4, EIF5B, ARHGEF15, RALGAPA1, MITF, DCK, ASRGL1, CLIP1, TRAF3, SRSF2, VGLL2, KPNB1, ARHGEF5, FKBP5, ATG4D, RGS16, SRSF1, RNF8, ZBTB42, LIN7C, STK4, NPAS2, ARHGAP12, FXR1, MAPK8, IRAK2, BTBD3, NLRP5, SHOC2, SRGAP3, HSPA9, RANBP3, MYEF2, SUCO, ARHGAP29, HSPBAP1, BICC1, MFHAS1, FER, CSDC2, CAPRIN1, TAOK1, WNK3, RLIM, AQP11, LSM14A |
| GO:0005654~nucleoplasm | 53 | 1.51E-07 | ZNF512B, DDX3X, NAA40, CDKN1B, ONECUT2, CDCA7L, RORB, ETS1, RASSF2, SMARCAD1, KPNA4, ZNF641, ZBTB37, WIPI2, MED4, POU3F1, RAD23B, DCK, SIRT1, ILF2, NAALADL2, TOX3, AFTPH, LCOR, CLOCK, KPNB1, ARHGEF5, FKBP5, ATG4D, GLYR1, MYRF, NUFIP2, RNF38, SRSF1, RNF8, ZBTB42, PDHB, NPAS2, CDKN2AIP, ATXN7, RAD21, UBN2, BEND3, SHOC2, ZNF148, BRD7, RANBP2, RANBP3, AGO3, SP1, KLF9, SNAI1, RLIM |
| GO:0000139~Golgi membrane | 10 | 0.013728 | LEPROT, GPR89A, SLC35A3, LMAN2, YIPF6, GCNT2, ZDHHC21, CLCN3, SORL1 |
| Molecular functions of bta-miR-199b | | | |
| GO:0000978~RNA polymerase II core promoter proximal region sequence-specific DNA binding | 27 | 3.27E-04 | ONECUT2, ZBTB42, ZNF23, RORB, ETS1, NPAS2, CDKN2AIP, RBBP4, ZNF704, ZNF527, ZKSCAN5, ZNF226, ZNF148, ZNF641, ZBTB18, ZFP2, ZBTB37, MITF, EVX2, POU3F1, SIRT1, SP1, KLF9, ZNF516, SNAI1, CLOCK, ZNF652 |
| GO:0000981~RNA polymerase II transcription factor activity, sequence-specific DNA binding | 24 | 0.001486 | ZBTB18, ZNF791, ONECUT2, MYRF, ZFP2, ZBTB42, MITF, EVX2, ZNF23, POU3F1, ETS1, NPAS2, CDKN2AIP, SP1, KLF9, ZNF527, ZNF516, ZNF614, ZNF579, ZKSCAN5, ZNF226, ZNF654, CLOCK, ZNF641 |
| GO:0003677~DNA binding | 20 | 0.008888 | DDX3X, ONECUT2, MYRF, ZFP2, MITF, EVX2, ILF2, DCK, NPAS2, EEPD1, TOX3, ZNF527, SMARCAD1, LCOR, ZNF226, ZNF654, ZNF148, PPARGC1A, SOS2, GLYR1 |
| GO:0003723~RNA binding | 19 | 0.002342 | RANBP2, RBM47, DDX3X, MYEF2, RBPMS, NUFIP2, SRSF1, HDLBP, PRPF40A, LARP4, BICC1, CDKN2AIP, AKAP1, PAN3, AGO3, CAPRIN1, AGO1, SRSF2, PPARGC1A |
| GO:0042803~protein homodimerization activity | 16 | 0.005013 | TGFB2, TENM4, FZD4, RNF8, ECE1, STK4, DCK, FXR1, TOX3, FLRT3, ACOX1, SP1, IRAK2, KIT, MYH9, MAP3K11 |
| KEGG pathway analysis for bta-miR-199b | | | |
| bta05200: Pathways in cancer | 17 | 0.00123 | RB1, TGFB2, CDKN1B, JAG1, FZD4, HHIP, IL23R, HGF, FZD6, MITF, STK4, ETS1, MAPK8, TRAF3, SP1, KIT, SOS2 |
| bta04014: Ras signaling pathway | 8 | 0.035763 | MAPK8, RALGAPA1, HGF, cKIT, SHOC2, STK4, ETS1, SOS2 |
| bta05224: Breast cancer | 7 | 0.011623 | RB1, JAG1, SP1, FZD4, KIT, FZD6, SOS2 |
| bta05226: Gastric cancer | 7 | 0.012725 | RB1, TGFB2, CDKN1B, FZD4, HGF, FZD6, SOS2 |
| bta04141: Protein processing in endoplasmic reticulum | 7 | 0.021462 | GANAB, MAPK8, LMAN2, DERL2, UBE2G1, RAD23B, SEC24C |

Supplementary Table S14: Sequence of the primers for miRNA target genes and reference genes

| miRNA | Primer sequence (5’-3’) | Length | Tm |
| --- | --- | --- | --- |
| bta-miR-195 | GGACCGCAGTAGCAGCACAG | 21 | 58 |
| bta-miR-1246 | GACGCGCAGAATGGATTTTTGGAG | 23 | 56 |
| bta-miR-339b | GACCAGTCCCTGTCCTCCAG | 20 | 58 |
| bta-miR-199b | GACGCCCAGTGTTTAGACTATCTG | 24 | 57 |
| bta-let-7a-5p | GACTGAGGTAGTAGGTTGT | 19 | 57.6 |
